# SHINE Transcription Factors Act Redundantly to Pattern the Archetypal Surface of Arabidopsis Flower Organs

**DOI:** 10.1371/journal.pgen.1001388

**Published:** 2011-05-26

**Authors:** Jian Xin Shi, Sergey Malitsky, Sheron De Oliveira, Caroline Branigan, Rochus B. Franke, Lukas Schreiber, Asaph Aharoni

**Affiliations:** 1 Department of Plant Sciences, Weizmann Institute of Science, Rehovot, Israel; 2 Department of Ecophysiology, University of Bonn, Bonn, Germany; 3 Department of Biology, University of York, York, United Kingdom; Peking University, China

## Abstract

Floral organs display tremendous variation in their exterior that is essential for organogenesis and the interaction with the environment. This diversity in surface characteristics is largely dependent on the composition and structure of their coating cuticular layer. To date, mechanisms of flower organ initiation and identity have been studied extensively, while little is known regarding the regulation of flower organs surface formation, cuticle composition, and its developmental significance. Using a synthetic microRNA approach to simultaneously silence the three SHINE (SHN) clade members, we revealed that these transcription factors act redundantly to shape the surface and morphology of Arabidopsis flowers. It appears that SHNs regulate floral organs' epidermal cell elongation and decoration with nanoridges, particularly in petals. Reduced activity of SHN transcription factors results in floral organs' fusion and earlier abscission that is accompanied by a decrease in cutin load and modified cell wall properties. SHN transcription factors possess target genes within four cutin- and suberin-associated protein families including, CYP86A cytochrome P450s, fatty acyl-CoA reductases, GSDL-motif lipases, and BODYGUARD1-like proteins. The results suggest that alongside controlling cuticular lipids metabolism, SHNs act to modify the epidermis cell wall through altering pectin metabolism and structural proteins. We also provide evidence that surface formation in petals and other floral organs during their growth and elongation or in abscission and dehiscence through SHNs is partially mediated by gibberellin and the DELLA signaling cascade. This study therefore demonstrates the need for a defined composition and structure of the cuticle and cell wall in order to form the archetypal features of floral organs surfaces and control their cell-to-cell separation processes. Furthermore, it will promote future investigation into the relation between the regulation of organ surface patterning and the broader control of flower development and biological functions.

## Author Summary

The cuticular layer that covers all aerial parts of plants plays a vital role not only in the interaction with environment but also in plant development and growth. Despite the recent significant achievements in the identification of structural genes involved in cuticle biosynthesis and secretion, little is known regarding the regulation of metabolic pathways generating cuticular constituents, more specifically wax and cutin. The Arabidopsis AP2-type transcription factor SHINE1/WAX INDUCER1 (SHN1/WIN1) was the first assigned regulator of a cuticle-related metabolic pathway; nevertheless, its mode of action and biological function remain uncertain due to redundancy with two additional clade members. Here, by co-silencing all three SHN clade members using an artificial microRNAs approach, we demonstrated that SHN transcription factors act redundantly in patterning reproductive organ surface, modulating processes associated with cell elongation, adhesion, and separation, which secure the proper function of these organs. It appears that SHN transcription factors act directly on downstream cutin and cell wall–modifying genes. These factors are likely part of the genetic network controlling floral organ development. Thus, SHN transcription factors link together cuticle assembly, cell wall remodeling, and flower development to form the archetypal surface of floral organs mediating plant reproduction through pollination and seed dispersal.

## Introduction

In contrast to other plant cell layers, the epidermis develops a unique cell wall that not merely constitutes of cellulose, hemicelluloses, pectins, and proteins but also of a cuticular matrix, which is largely composed of cutin embedded and overlaid with waxes [1]. Cutin, an insoluble cuticular polymer, is largely composed of interesterified hydroxy and hydroxy epoxy fatty acids and is attached to the outer epidermal layer of cells by a pectinaceous layer [2]. As the epidermal cell grows, the cuticle merges gradually with the cell wall components [3]. Although the role of the epidermis layer in regulating organ growth has remained controversial [4]–[5], it is clear that it is vital for plant survival, development and the interaction with the environment [6]–[7]. Cutin and wax are synthesized exclusively in the epidermis [8] and a massive flux of lipids occurs from the sites of lipid synthesis in the plastid and the endoplasmic reticulum (ER) to the plant surface during cuticle deposition [9]. Significant progress has been made over the past decade in identifying genes involved in the biosynthesis and secretion of cuticular lipids [10]–[11] and in the metabolism and assembly of primary cell wall components [12]–[14]. Despite the close connection between the cell wall and the cuticular matrix, mutants and phenotypes in one of these processes were rarely examined for alteration in the other. Furthermore, to our knowledge, co-regulation of these two processes at the molecular genetic level was overlooked up to now.

Biosynthesis of plant cuticle components and their secretion to the extracellular matrix involve the coordinated induction of several metabolic pathways, in which transcription factors may play a key role [9], [15]. The Arabidopsis SHINE1/WAX INDUCER1 (SHN1/WIN1) AP2-domain protein was the first transcription factor reported to control metabolic pathways generating cuticular waxes [16]–[17]. A subsequent study [18] indicated that SHN1/WIN1 controls cuticle permeability by regulating the expression of cutin biosynthesis genes, particularly *LACS2* (*LONG CHAIN ACYL-COA SYNTHETASE* 2). The induction of wax formation in leaves by over expression of individual *SHINE* clade genes was suggested to be a second step, possibly an indirect process following cutin biosynthesis [18]. Nevertheless, our current knowledge is limited with respect to the SHN1/WIN1 protein's mode of action and the involvement in particular developmental processes.

Arabidopsis SHN1/WIN1 transcription factor belongs to a small distinct clade of three proteins [16]. They all share two unique conserved motifs outside the AP2 domain, and all three proteins display the same shiny phenotype upon overexpression, suggesting their functional redundancy in cuticular lipid biosynthesis. Additional evidence for functional redundancy among the SHN clade members in cuticular lipid biosynthesis was provided by silencing *SHN1*/*WIN1* [18]. In these plants, floral morphology was not altered and the subtle reduction in the levels of cutin detected in entire flower extracts was enhanced in isolated petals. Besides, their notable expression patterns in reproductive organs suggested that they are probably redundant in function. The expression of *SHN1*/*WIN1* and *SHN3* overlapped in various flower organs including in the abscission zones while *SHN2* and *SHN3* were both expressed in the silique dehiscence zones. Interestingly, expression of *SHN2* was very specific to cell separation regions in the anthers and siliques. These expression profiles indicated that SHN transcription factors may also act in a combinatorial manner to secure reproductive organ development, protecting the exterior layers of the plants from environmental stresses. On the other hand, these three clade members differ in their spatial and temporal expression patterns, which suggests that each of them may play specific roles in various organs or under different conditions, and that the actual redundancy between the SHN factors is most probably in their target genes [16]. Further elucidation of the mode of SHN action, their target genes, and their precise connection to plant cuticle formation and plant development requires in-depth characterization of the SHN clade factors, which can be achieved by using double, possibly triple mutants to eliminate redundant activities [16]–[18]. In contrast to Arabidopsis, mutation in the barley *SHN1*/*WIN1* ortholog (*Nud*) was sufficient to generate a severe morphological change in which the typically hulled caryopses developed into naked ones [19]. Nud was suggested to direct the deposition of a lipidic matter on the pericarp epidermis that adheres the hull to the caryopsis in a way similar to postgenital fusions displayed by numerous cuticular mutants [20]–[21].

In this study we have co-silenced the three *SHN* clade members in order to decipher their modes of action and resolve their biological roles. We revealed that *SHN* clade genes regulate the elongation and decoration (i.e. nanoridges formation) of reproductive organ epidermal cells, particularly in the petal surface. They also emerge as mediators of cell adhesion and separation during abscission and dehiscence. Additionally, the results suggest that beside their function in the cutin pathway, these transcription factors possess putative downstream target genes that are involved in cell wall configuration through pectin modifying enzymes and structural proteins. Thus, the study of SHN transcription factors provides novel insight to the transcriptional control that mediates the patterning of reproductive organs surfaces and their associated separation processes in between cell layers.

## Results

### Co-silencing of the three *SHINE* clade genes results in severe morphological and surface phenotypes in floral organs

**Figure 1:**
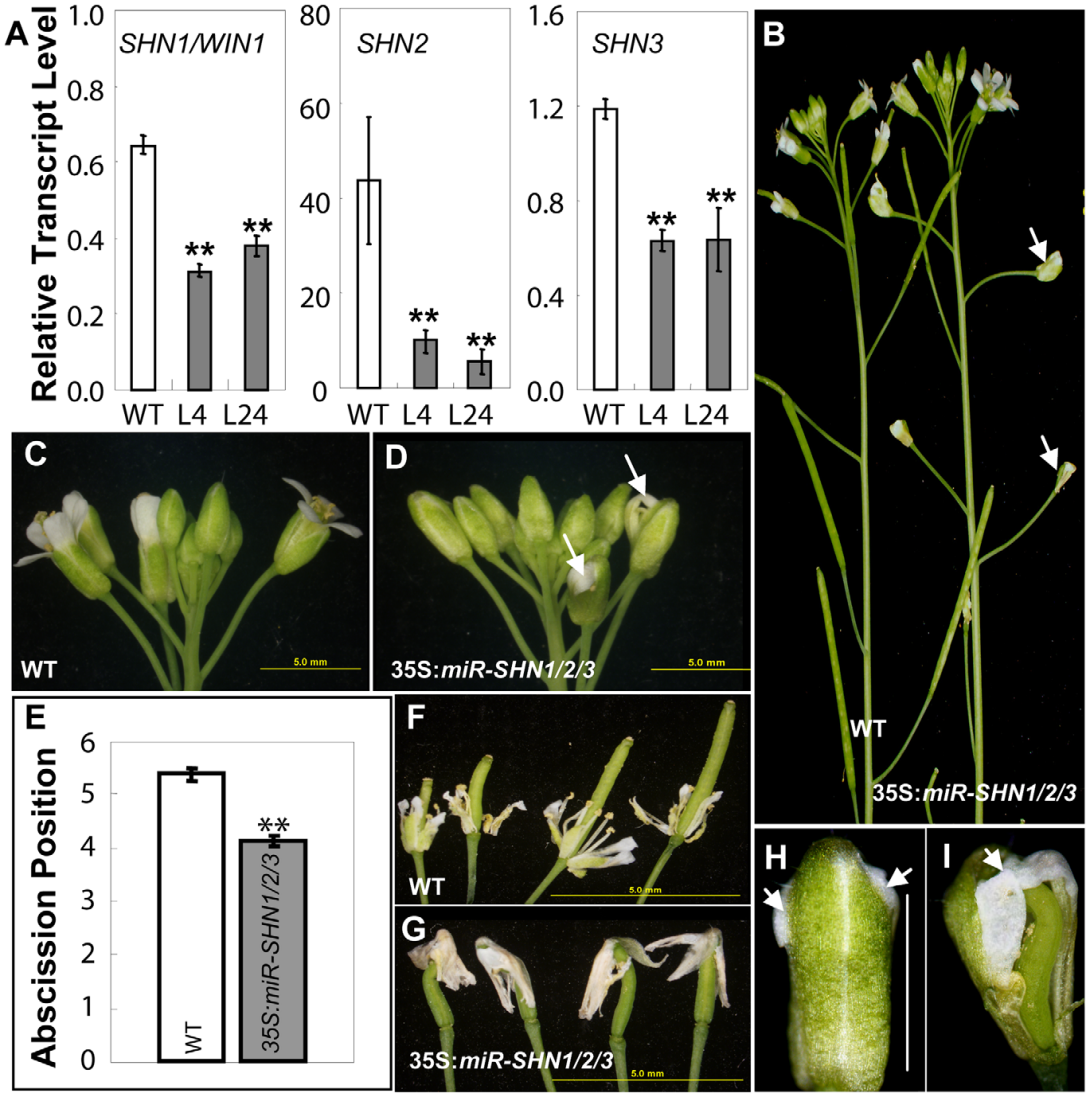
Co-silencing of the three *SHN* clade genes impacts reproductive organ morphology and cell type–specific characteristics. (A) Co-silencing of the three *SHN* genes as determined by real time RT-PCR in 35S*:miR-SHN1/2/3* young buds (n = 3). (B) 35S*:miR-SHN1/2/3* plants developed semi-sterile siliques (arrows). (C–D) 35S*:miR-SHN1/2/3* inflorescences displayed abnormal buds (arrows). (E) 35S*:miR-SHN1/2/3* floral organs (n = 50) abscised earlier than WT ones (n = 34). (F) Abscising WT flower organs. (G) Abscised 35S*:miR-SHN1/2/3* flower organs remain attached to the siliques. (H–I) An unopened bud and a bud with sepals and petals removed of 35S*:miR-SHN1/2/3* flowers, respectively. Arrows indicate organ fusion sites. In (A) and (E), means and standard errors are presented (**, p<0.01, Student's t-test).

To circumvent the likely functional redundancy between the Arabidopsis SHN clade members we generated plants in which they were simultaneously silenced through an artificial microRNA approach (Figure S1A and Text S1). The presence of cleaved products and transcriptional downregulation of all three *SHN* genes was confirmed in the 35S*:miR-SHN1/2/3* plants (Figure 1A and Figure S1B–S1C). No visual change was observed in these plants during vegetative growth and cuticle permeability of their rosette leaves was normal (Figure S1D–S1G). However, reproductive organs, particularly petals, were severely affected (Figure 1C–1D). This was evident already in buds that displayed postgenital fusions between petals and other floral organs at their tops (Figure 1H–1I). The expansion of petals and elongation of the carpels were restrained and they were curved and/or twisted (Figure 1I and Figure S1L–S1M). The changes in flower organ morphology also impinged on self-pollination and semi-sterility was occasionally detected (Figure 1B). Interestingly, mutant flower organs abscised earlier (Figure 1E and Figure S1J–S1K), and in some cases the abscised flower parts stayed attached to the top of the silique due to the postgenital organ fusion between them (Figure 1F–1G).

**Figure 2:**
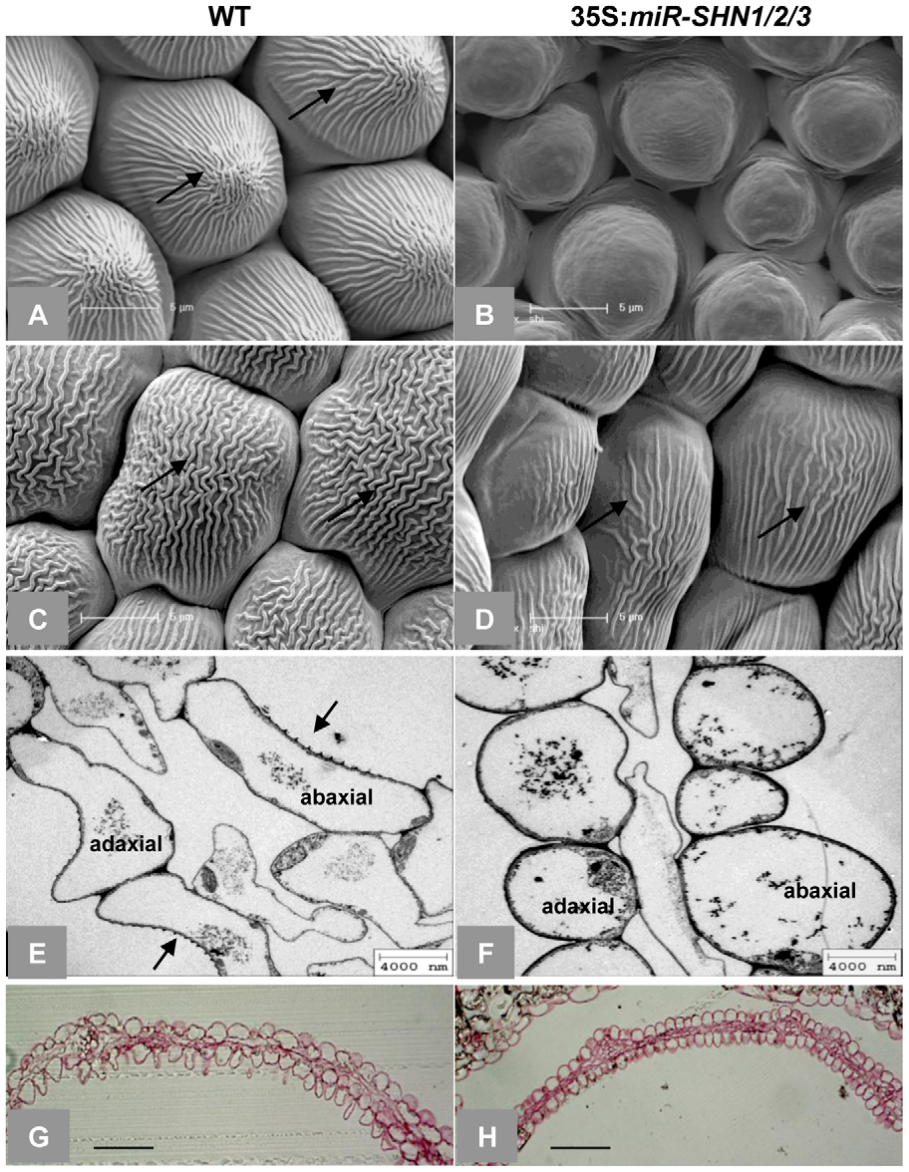
Changes in petal morphology and surface characteristics as observed with electron microscopy. (A–D) SEM images of adaxial (A–B) and abaxial (C–D) petal epidermis, respectively, arrows indicate nanoridges. (E–F) TEM images of petal cross sections, arrows indicate nanoridges. (G–H) Light microscopy images of Rethinium Red stained petal cross sections. Scale bars: A–D: 5 µm; E–F: 4 µm; G–H: 50 µm.

Microscopic observation of floral organs surfaces in the 35S:*miR-SHN1/2/3* plants revealed extensive alterations to their archetypal epidermal cells (Figure 2 and Figure S2). Both abaxial and adaxial conical cells of petals appeared less elongated, more spherical and compact in addition to being separated with wider spaces as compared to the wild-type (WT) cells (Figure 2). Remarkably, nanoridges, typically displayed on WT petal epidermis [22]–[23], were either absent (adaxial) or significantly reduced (abaxial) in the 35S*:miR-SHN1/2/3* petal cells (Figure 2A–2F). Altered epidermis cell size, shape and nanoridge decoration was also observed in surfaces of additional floral organs such as sepals, styles, filaments, nectaries, and pedicles (Figure S1N–S1Q and Figure S2). The observed phenotypes provided evidence that the *SHN* clade genes function redundantly in cell elongation, separation and nanoridge formation of reproductive organs. In contrast to the 35S*:miR-SHN1/2/3* floral organs, silencing *SHN1/WIN1* alone did not cause any visible morphological changes in floral organs, particularly in petal surfaces (Figure S3).

### Cutin and cell wall–related genes are likely downstream targets of SHINE transcription factors

In order to unravel the molecular mechanism by which the SHN factors regulate the patterning of reproductive organ surfaces we compared the transcriptome of 35S:*miR-SHN1/2/3* flower buds to the one of WT. A modest set of 38 differentially expressed genes was detected; 30 transcripts including *SHN1* and *SHN3* (*SHN2* was not represented in the array) were downregulated while 8 others were upregulated in 35S*:miR-SHN1/2/3* buds (Table 1).

**Table 1:** A list of genes that displayed up- or down-regulated expression in flower buds of the 35S:*miR-SHN1/2/3* plants as compared with wild-type ones.

	Gene	Anotation	Functional	Fold†	Public Gene Expression Data‡								
Locus*	Product		Category	Change	Pe	Dp	Up	Ds	Us	Ne	Sa	C1	C3
Down-Regulated Genes													
At4g13210	*PLL23*	Pectate lyase	Cell wall structure	−3.46	+	+					+	+	
**At4g28160**	*HPRP*	Hydroxyproline-rich glycoprotein	Cell wall structure	**−3.43**	+	+						+	
**At5g55720**	*PLL14*	Pectate lyase	Cell wall structure	**−3.05**	+	+						+	
**At2g05540**	*GRP*	Glycine-rich protein	Cell wall structure	**−1.67**			+		+				
**At1g58430**	*RXF26*	GDSL-motif lipase/hydrolase	Lipid metabolism	**−3.30**	+	+					+	+	+
**At1g63710**	*CYP86A7*	Cytochrome P450	Lipid metabolism	**−2.81**	+	+					+	+	+
**At4g24140**	*BDG3*	Hydrolase	Lipid metabolism	**−2.81**	+	+			+				
**At2g42990**		GDSL-motif lipase/hydrolase	Lipid metabolism	**−2.25**	+	+					+	+	
**At1g01600**	*CYP86A4*	Cytochrome P450	Lipid metabolism	**−2.03**	+	+					+	+	
**At5g33370**		GDSL-motif lipase/hydrolase	Lipid metabolism	**−1.54**	+	+					+	+	+
**At5g22500**	*FAR1*	Fatty acid reductase	Lipid metabolism	**−1.53**					+	+	+		
**At3g11480**	*BSMT1*	SAM:carboxyl methyltransferase	Methionine metabolism	**−5.51**									
**At2g26400**	*ARD3*	Acireductone dioxygenase	Methionine metabolism	−1.69					+				
**At3g62950**	*GRXC11/ROXY4*	Glutaredoxin-C	Redox regulation	−1.98									
**At5g05250**	*PRX02*	Peroxidase	Redox regulation	−1.94					+				
**At5g23970**		Acyl transferase	Secondary metabolism	**−2.03**		+					+	+	
**At5g60090**		Protein kinase	Signaling	**−1.54**									
At4g08850		Receptor like kinase	Signaling	−1.50			+		+				
**At5g03350**		Receptor like protein	Signaling	−3.32					+				
At1g52690	*LEA*	Late embryogenesis abundant protein	Stress response	**−3.17**		+			+	+			
**At2g43620**		Chitinase	Stress response	−1.80									
**At4g14365**		C3HC4-type RING finger	Transcription factor	**−1.53**			+		+				
**At1g15360**	*SHN1/WIN1*	ERF/AP2 transcription factor	Transcription factor	**−3.47**	+	+				+	+		
**At5g25390**	*SHN3*	ERF/AP2 transcription factor	Transcription factor	**−1.57**		+		+					
At4g33530	*POT13/KUP5*	Potassium transporter	Transport	**−1.90**									
**At1g27940**	*PGP13/MDR15*	Multidrug resistance P-glycoprotein	Transport	**−1.57**	+	+						+	
**At1g22690**		Gibberellin-responsive protein	Unknown	**−2.09**									
At3g56260		Expressed protein	Unknown	−1.83	+	+							
At4g27450		Expressed protein	Unknown	−1.72					+				
At2g16760		Expressed protein	Unknown	**−1.55**	+	+							
Up-Regulated Genes													
At1g70720	*PMEI*	Pectin methylesterase inhibitor	Cell wall structure	**1.52**	+						+	+	
**At3g57510**	*ADPG1*	Polygalacturonase	Cell wall structure	**2.33**					+				
**At4g15210**	*BAM5/RAM1*	Beta-amylase	Starch metabolism	1.56		+		+					
**At3g28740**	*CYP81D11*	Cytochrome P450	Stress response	1.59			+	+					
At5g43510		Defensin-like (DEFL) family	Stress response	2.14									
At1g58270		ZW9 mRNA	Unknown	1.56									
At5g22970		Expressed protein	Unknown	1.71									
At3g56610		Expressed protein	Unknown	1.98									

*Promoters of the bold genes were successfully cloned and subsequently used for transient assay and underlined ones were activated by at least one of these three SHN transcription factors;

**†:** Gene expression of the bold genes, together with that of *SHN2*, were validated with real-time RT-PCR analysis (Figure S4);

**‡:** *In silico* analysis of the expression patterns of these differentially expressed genes. Pe, Petal-specific genes (GeneVestigator, [35]); Dp and Up, Genes down-and up-regulated in senescing petals, respectively [36]; Ds and Us, Genes down-and up-regulated in senescing siliques [36], respectively; Ne, Genes enriched in nectary [38]; Sa, Genes enriched in stamen abscission zones [37]; C1 and C3, Genes co-expressed with *SHN1/WIN1* and *SHN3*, respectively.

Interestingly, one of the two main functional categories that dominated the differential genes represented six cell wall related genes (Table 1). Four of them corresponded to enzymes associated with pectin degradation or modification, including two pectate lyases (*PLL14* and *PLL23*), a polygalacturonase (*ADPG1*) and a pectin methylesterase inhibitor (*PMEI*). Two additional genes putatively encode cell wall structural proteins: a hydroxyproline-rich glycoprotein (*HRGP*) and a glycine-rich protein (*GRP*). The second major category consisted of seven genes that putatively encode cuticular lipids (mainly cutin) related proteins, including 2 cytochrome P450s (*CYP86A4* and *CYP86A7*) implicated in flower cutin biosynthesis [18], [23], three GDSL-motif lipase/hydrolases (*RXF26*, *At2g42990*, and *At5g33370*) that are highly similar to the reported cutin related lipase *At2g04570* [18], and one hydrolase (*BODYGUARD 3*, *BDG3*), the closest homolog of *BDG1*, an epidermis-specific extracellular protein associated with cuticle formation [24]. *Fatty Acyl-CoA Reductase 1 (FAR1)*, the seventh gene was associated with primary fatty alcohol production [25]; its additional and/or alternative function with relation to surface lipids will be discussed below.

Two downregulated genes encoded a potassium transporter (*KUP5*) and an ABC transporter (*PGP13/MDR15*); both are involved in cell growth [26]–[28]. Additional three downregulated genes encoded kinase and/or kinase like proteins, that are potentially involved in reporting sensing aspects of cell wall structure and function [29]. Differential expression of 24 genes including the three *SHN* genes was subsequently validated using realtime RT-PCR assays (Figure S4 and Text S1). Altogether, gene expression analysis results indicated that the phenotype observed in 35S:*miR-SHN1/2/3* reproductive organs probably result from the altered expression of their target genes, particularly those related to cutin and cell wall remodeling and function.

### Silencing SHINE clade genes reduces flower cutin load and modifies petal cell wall structure

**Figure 3:**
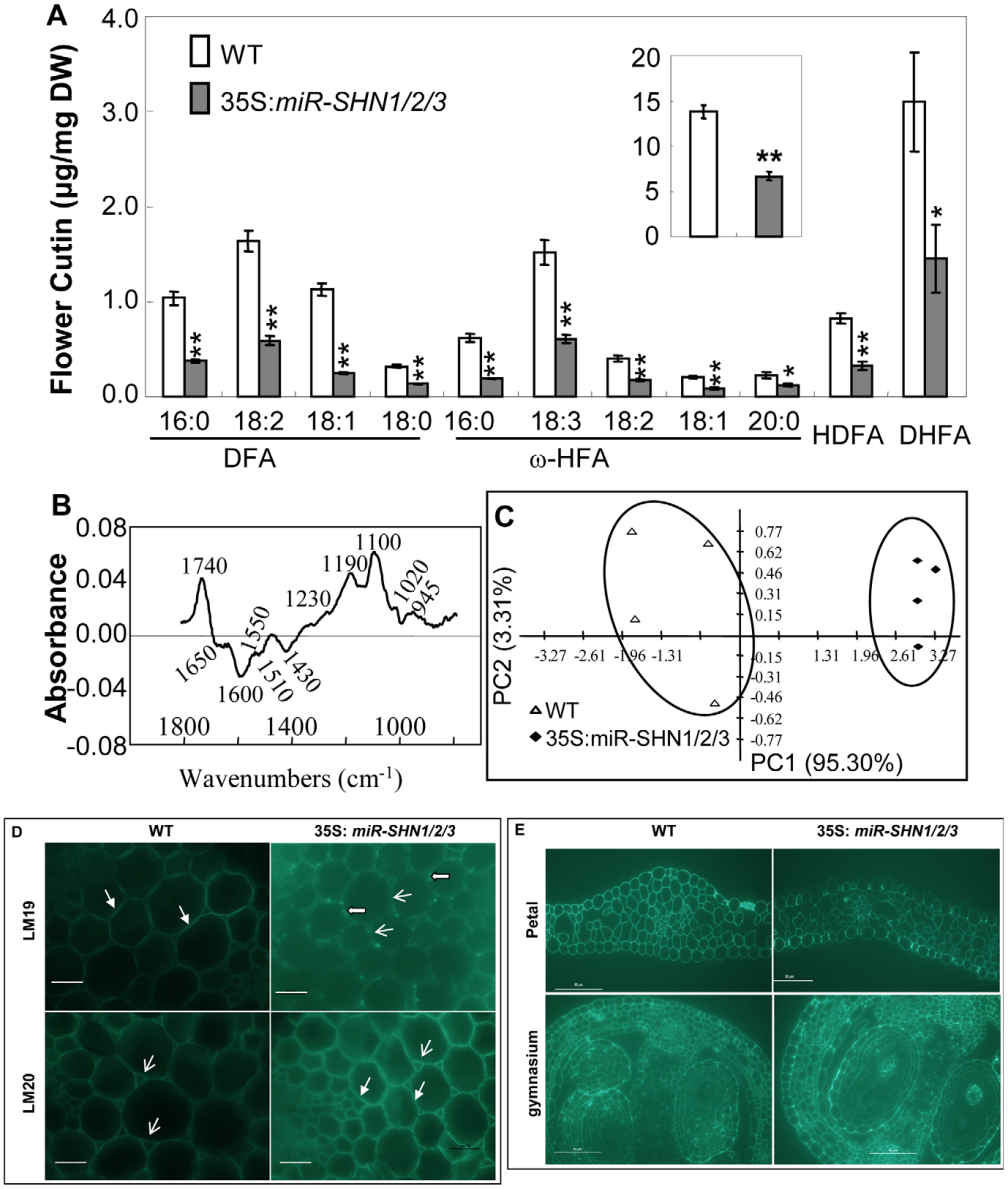
Altered flower cutin monomers levels and petal cell wall structure in 35S*:miRSHN1/2/3* plants. (A) Cutin monomer content in flowers of WT and 35S*:miRSHN1/2/3* plants (total flower cutin in the insert). FA, fatty acids; DFA, α,ω-dicarboxylic FA; 2-HFA, 2-hydroxy FA; ω-HFA, ω-hydroxy FA; HDFA, C16-9/10-hydroxy DFA; DHFA, C16-9/10,16-HFA. Values are means and standard errors (n = 3; *, p<0.05; **, p<0.01, Student's t-test). (B–C) Comparison of 35S*:miR-SHN1/2/3* and WT petal cell wall by FTIR analysis. In (B), difference spectra obtained by digital subtraction from the WT average spectrum of the 35S*:miR-SHN1/2/3* average spectrum, peaks below and above the zero are enriched in the 35S*:miR-SHN1/2/3* and WT, respectively. In (C), principle component analysis (PCA) displaying the separation of WT and 35S*:miR-SHN1/2/3* FTIR spectra. (D–E) Indirect immunofluoresence detection of the localization of monoantibodies LM19 and LM20 to cell walls of transverse sections of WT and 35S:*miR-SHN1/2/3*. D. Hand section of inflorescence stems. The fluorescence of LM19, localizing pectin to the middle lamella, became weaker but aggregated along the middle lamella in the *35S:miR-SHN1/2/3* line; the fluorescence of LM20, localizing pectin to the air spaces, became stronger not only in the air spaces but also in the middle lamella in the *35S:miR-SHN1/2/3* line. E. Microtome section of petals and the gynoecium. The fluorescence of LM20 became stronger in petals and the developing seed coats in the *35S:miR-SHN1/2/3* line. Head-filled arrows indicate meddle lamella, arrows point to air spaces, and block arrows designate aggregations.

Because plant organ fusion and separation have been reported to be associated with cuticle [19]–[20], [22], we subsequently examined the changes in cuticular lipids in leaf and flower tissues of the 35S*:miR-SHN1/2/3* plants. While the amount of leaf cutin was not significantly changed (Figure S5A), the amount of flower cutin in the 35S*:miR-SHN1/2/3* plants was reduced to 48.4% of the wild-type (Figure 3A). The changes in flower cutin loads reflected the changes in the cuticle permeability in flower tissues (Figure S1F–S1I). The substantial decrease of dioic acids (DFA, particularly C_16_, C_18:2_ and C_18:1_), ω-hydroxy fatty acids (ω-HFA, particularly C_16_ and C_18:3_), 9/10,16-dihydroxy hexadecanoic acid (C_16_-9/10,16-DHFA) and 9(10)-hydroxy-hexadecanedioic acid (C_16_-9/10-HDFA) largely contributed to the reduced flower cutin in the 35S*:miR-SHN1/2/3* plants. Levels of cuticular waxes in either leaves or flowers were not significantly altered in the 35S*:miR-SHN1/2/3* lines (Figure S5B–S5C).

The finding that co-silencing the three *SHN* genes affected the expression of pectin modifying genes prompted us to analyze the cell wall pectin composition in the seed mucilage and buds. GC-MS analysis did not reveal any significant compositional changes in seed mucilage and the bud cell wall pectic monosaccharides (Figure S5D–S5E and Text S1). We next used Fourier transform infrared (FTIR) spectroscopy to examine if petals of the 35S*:miR-SHN1/2/3* plants exhibited structural changes in their cell walls. Principal component analysis (PCA) showed a clear separation of the petal FTIR spectra between 35S:*miR-SHN1/2/3* petals and WT ones (Figure 3C). The difference spectrum (Figure 3B) generated by digitally subtracting the average 35S*:miR-SHN1/2/3* spectrum from the average WT petals spectrum showed that WT petal cell wall had more acyl esters (1740 cm^−1^) [30]–[31], amide III proteins (1230 cm^−1^) [32], and non-cellulosic carbohydrates (1100 to 900 cm^−1^) [33]. In contrast, 35S:*miR-SHN1/2/3* petal cell walls contained more salt-form of pectin (1430 and 1600 cm^−1^, respectively) [32], amide I and amide II proteins (1650 and 1550 cm^−1^, respectively) [32]–[33], and phenolic esters or aromatic lignins (1635 and 1510 cm^−1^) [32]–[33].

To localize the pectic polysaccharides in the cell walls, two novel rat monoclonal antibodies LM19 and LM20, which recognize pectic homogalacturonan (HG) epitopes [34], were used to hybridize transverse sections of inflorescence stems (pith parenchyma) and flowers. Similar to an earlier observation in tobacco plants [34], LM19 localized pectin to junctures (middle lamella) while LM20 localized pectin to the intercellular spaces (air spaces) in both WT and 35S:*miR-SHN1/2/3* inflorescence stems (both antibodies appeared as green fluorescence) (Figure 3D). However, the florescence of LM19 in transverse sections of the 35S:*miR-SHN1/2/3* samples became weaker and they were aggregated along the middle lamella line. Moreover, the florescence of LM20 in 35S:*miR-SHN1/2/3* was enhanced not only in the air spaces but also in the middle lamella. In addition, the florescence of LM20 binding to air spaces become stronger in microtome sections of 35S:*miR-SHN1/2/3* petals and developing seed coats, as compared to WT ones (Figure 3E). Because the binding of both LM19 and LM20 to pectin is sensitive to pectate lyase treatment and they bind preferably to HG [35], these results indicated alteration to HG distribution in the mutants. Therefore, silencing the *SHN* clade genes not only affected the cutin matrix of the cuticle but also the cell wall matrix of the cell.

### Characterization of the putative SHINE transcription factors target genes

Remarkably, *in silico* analysis (Table 1) showed that as *SHN1*/*WIN1*, 13 of the differentially expressed genes (12 downregulated and one up regulated in the 35S*:miR-SHN1/2/3* plants) display a petal-specific expression pattern [35]. Moreover, all those 12 petal-specific downregulated genes, together with *SHN1/WIN1*, *SHN3*, and 3 more genes display decreased expression in senescing petals [36]. Furthermore, 9 of the differential genes in addition to *SHN1/WIN1* are expressed in the stamen abscission zone (AZ) [37] while 2 genes and *SHN1/WIN1* are enriched in the nectary [38], and 13 genes and *SHN3* are differentially expressed in senescing siliques [36]. These results provided evidence that both the *SHN* factors and their putative targets are associated with reproductive organ development (i.e. petals and siliques) and possibly cell separation as well. The series of genes altered in the 35S:*miR-SHN1/2*/3 plants were also strongly co-expressed with the *SHN* factors (Figure S6 and Table S2), further indicating the functional link between the groups of genes we have identified in the array analysis.

**Figure 4:**
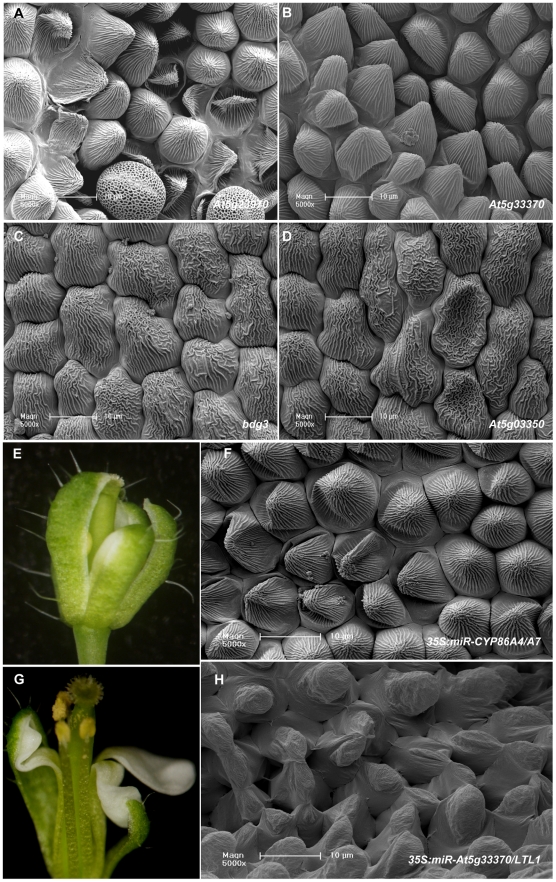
Putative downstream target genes of SHN transcription factors in patterning floral organs. (A–D) SEM images of defective petal epidermis observed in knock-out mutants of four putative SHN transcription factors target genes: A and B, Adaxial epidermis of knock out lines of *At5g23970* (a BAHD family member acyltransferase) and *At5g33370* (a GDSL-motif lipase/hydrolase), respectively; (C) and (D), Abaxial epidermis of *bdg3* (*At4g24140*; a hydrolase) and *rlp* (*At5g03350*; a legume lectin protein), respectively. See images of WT petal epidermis in Figure 2 and Figure S7. (E–F) Phenotypes observed in 35S*:miR-CYP86A4/A7* (cutin-related cytochrome P450s) transgenic plants: (E) A bud showing the fusion between petals and sepals and (F) Collapsed adaxial petal epidermis cells. (G–H) Phenotypes observed in 35S*:miR-At5g33370/At3g04290* (GDSL-motif lipase/hydrolases) transgenic plants: (G) A flower showing fused and folded petals and (H) Abnormal adaxial petal epidermis cells.

In order to examine whether loss of function of the putative SHN clade proteins target genes results in alteration to petal surface we screened for T-DNA insertions in the entire set of 28 downregulated genes. Homozygous knockout lines could be identified for thirteen of them and their petals surface was examined using scanning electron microscopy (Figure S7). Petals of the *At5g23970* (a putative acyltransferase) and *At5g33370* (a putative GDSL-lipase) knockout plants exhibited collapsed conical cells, while those of *At4g24140* (*bodyguard3/bdg3*), *At5g03350* (a receptor like protein) and *At1g01600* (*cyp86a4*) displayed abnormal abaxial nanoridges (Figure 4A–4D).

Some differential genes identified in microarray analysis belong to large multi-gene families as for example lipases and cytochrome P450s. This suggested that they might be functionally redundant with other family members. We therefore co-silenced the *CYP86A4* with *CYP86A7*, and the GDSL-lipase *At5g33370* with its closest homolog *At3g04290*, *LTL1* [39], via the artificial microRNA method. Plants co-silenced for either one of these pairs of genes displayed severe floral organ fusion and alteration in the conical cell shape and/or epidermis cell decoration (Figure 4E–4H). These results from single knockouts and the co-silenced lines provided additional evidence for the functional link between the putative SHN proteins target genes and the patterning of the petal surface.

### SHINE proteins activate promoters of their putative target genes

**Figure 5:**
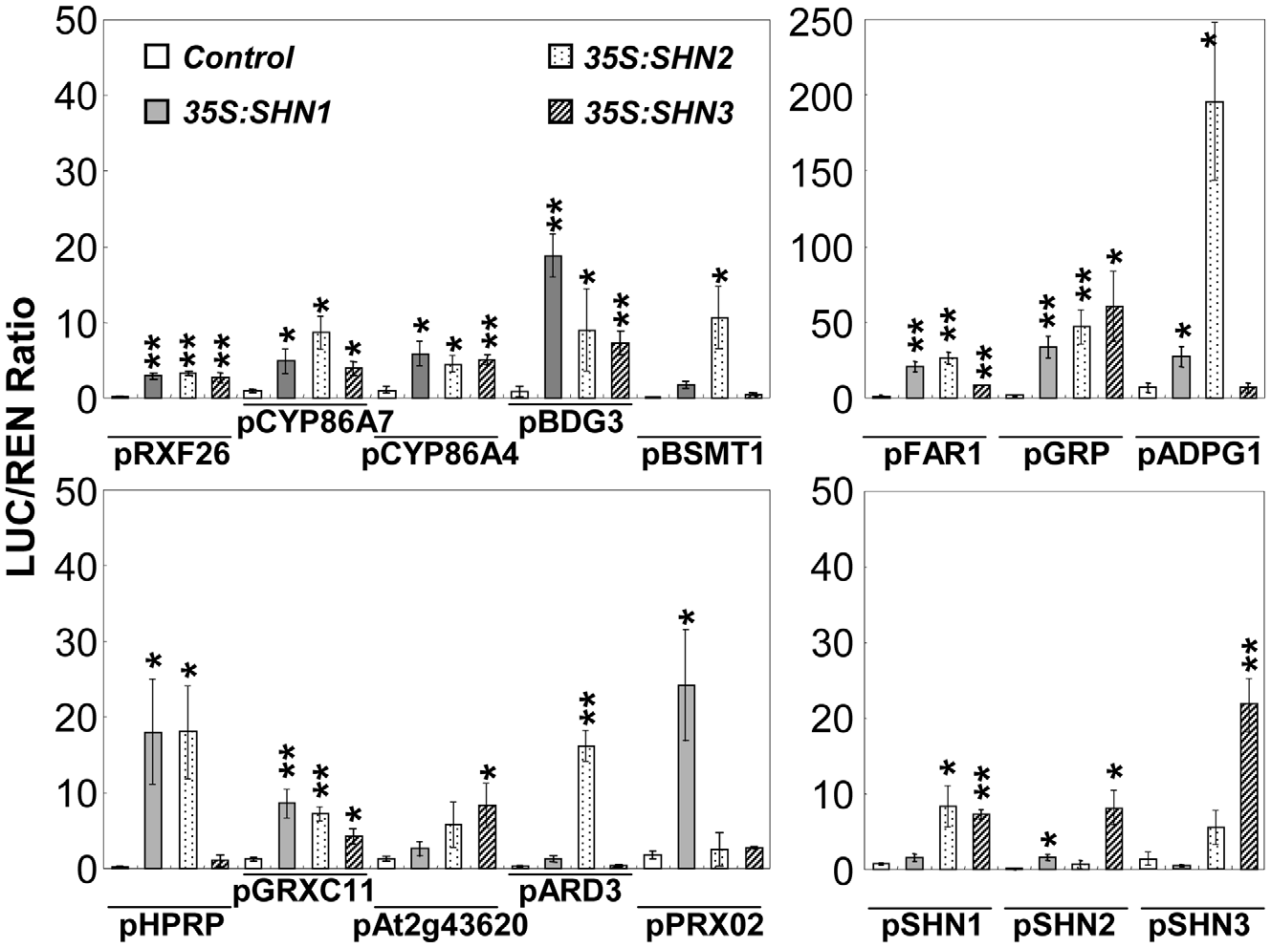
Transient expression assays of SHN transcription factors putative target gene promoter regions. Those promoter regions were co-infiltrated with plasmids containing *SHN* transcription factors fused to the 35S promoter. Promoters of *SHN* genes were also included. LUC/REN (firefly luciferase/renilla Luciferase) values represent means and standard errors (n = 4; *, p<0.05; **, p<0.01, Student's t-test).

We subsequently examined the activation of promoters of genes that were differentially expressed in the 35S:*miR-SHN1/2*/3 plants by the SHN transcription factors using a dual luciferase assay system [40]. Promoter regions of 23 putative targets and the 3 *SHN* clade genes were examined. Thirteen out of 23 were significantly activated by at least one of the three SHN transcription factors (Figure 5). Promoter regions of seven genes were activated by all three factors including the ones of *RXF26*, *CYP86A4*, *CYP86A7*, *BDG3*, *FAR1*, *GRP*, and *GRXC11*. The promoters of *PRX02* (a peroxidase), *ARD3* (an acireductone dioxygenase), and *At2g43620* (a chitinase) were only activated by SHN1/WIN1, SHN2, and SHN3, respectively. Interestingly, SHN1/WIN1 and SHN2 were able to activate each other's promoter, while SHN3 was able to activate all three *SHN* genes promoters. We included *LACS2* promoter as a positive control [18], however, activation of this gene promoter by the SHN transcription factors was not detected in our assay. These results further confirmed the functional redundancy of SHN transcription factors in cuticle and cell wall metabolism by acting directly on common targets and by regulating each other and possibly their own transcription.

### GA modulates the expression of *SHINE* clade genes

Gibberellins (GAs) are a class of plant hormones involved in the regulation of flower development in Arabidopsis. GA promotes the expression of floral homeotic genes *APETALA3* (*AP3*), *PISTILLATA* (*PI*), and *AGAMOUS* (*AG*) by antagonizing the effects of DELLA proteins, thereby allowing continued flower development [41]. Publically available array data suggested that GA promotes the expression of *SHN1/WIN1* while DELLA suppresses *SHN1/WIN1* expression, which was examined in the *ga1-3* and the *ga1-3 gai-t6 rga-t2 rgl1-1 rgl2-1* (i.e. *penta*) [35]. Remarkably, in young flower buds, GA promotes the expression of thirteen of the putative *SHN* target genes identified in this study while it down regulates the expression of another four putative target genes, all of them in a DELLA dependent manner ([42], Figure S8A–S8B). In addition, GA regulates another two putative *SHN* target genes, AT4G27450 and AT1G27940, in a DELLA-independent way [42]. The results described above led us to suggest that GA might be involved in cuticle assembly during flower organ development via modulating the expression (directly or indirectly) of the *SHN* transcription factors and their downstream target genes.

**Figure 6:**
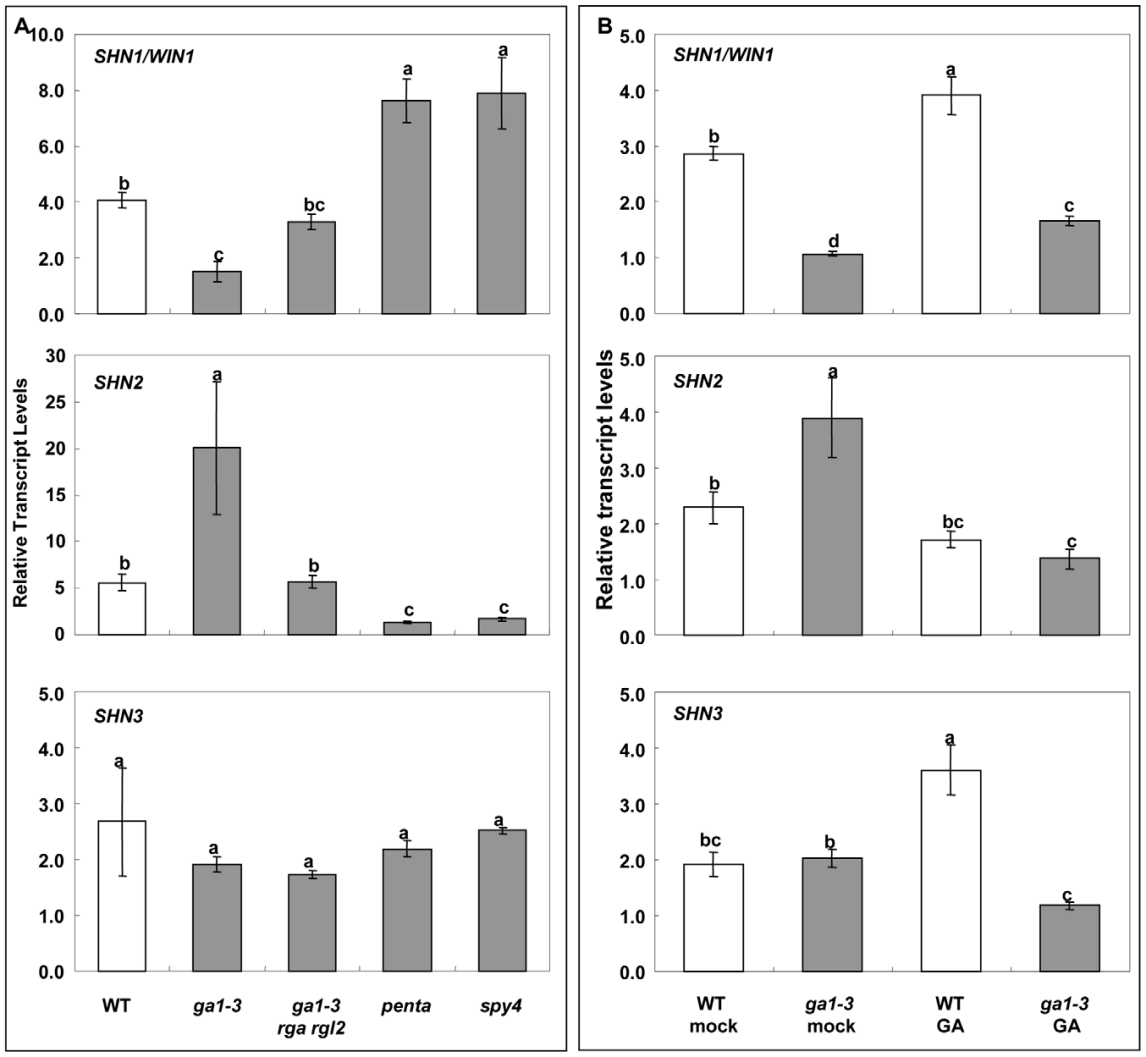
Giberellin (GA) biosynthesis and signaling affects the expression of *SHN* clade genes in flower buds. (A) Expression of *SHN* clade genes in GA biosynthesis and signaling mutants; (B) Expression of *SHN* clade genes as affected by the application of 100 µM exogenous GA. Values represent means and standard errors (n = 3). Different letters between different columns indicate the level of significance (p<0.05) obtained with a Student's t test. *ga1-3* (GA biosynthesis mutant); *rga rgl2*, double DELLA mutant (RGA, repressor of *ga1-3*; RGL2, RGA like 2) in the *ga1-3* background; *penta*, *gai-t6 rga-t2 rgl1-1 rgl2-1* in the *ga1-3* background; *spy4* (*spindly4*) (repressor of GA signaling).

To test this assumption, we examined the expression of *SHN* genes in different GA biosynthesis or signaling mutants (Figure 6A). Quantitative RT-PCR analysis showed that expression of *SHN1/WIN1* is downregulated in the *ga1-3* mutant that is defective in GA biosynthesis. It also showed that DELLA significantly suppressed *SHN1/WIN1* expression, since the expression of *SHN1/WIN1* in the double (*rga-t2 rgl2-1*; partial loss of DELLA signaling) and quadruple DELLA (*gai-t6 rga-t2 rgl1-1 rgl2-1*) mutants in the *ga1-3* background was recovered to equal and even much higher levels than that of the wild type, respectively. Knockout of SPY4, another repressor of GA signaling, also enhanced *SHN1/WIN1* expression as compared to the wild type. As compared to *SHN1/WIN1*, *SHN2* showed the opposite expression pattern in the background of the various GA biosynthesis and signaling mutants. Expression of *SHN2* was upregulated in the *ga1-3* background while it was significantly downregulated in the *penta* and *spy4* mutant backgrounds. Interestingly, neither GA biosynthesis nor the signaling mutants significantly altered *SHN3* expression.

We also examined the expression of *SHN* clade genes in both the WT and *ga1-3* flower buds in response to exogenous GA application (Figure 6B). Quantitative RT-PCR analysis showed that GA application to the *ga1-3* mutant increased the levels of *SHN1/WIN1* and decreased the levels of *SHN2* expression as compared to *ga1-3* alone, as does the endogenous GA (Figure 6A). The response of *SHN3* might be different between endogenous and externally applied GA as its expression did not change significantly in the *ga1-3* background alone while it was altered upon GA supplementation in either the WT or *ga1-3* (Figure 6B).

Finally, we also carried out GC-MS analysis of the flower cuticular lipids of the GA biosynthesis and signaling mutants. While flower waxes were not significantly altered in the *ga1-3* and *penta* mutant flowers, the total cutin load, particularly of the 9/10,16-dihydroxy hexadecanoic acid (C16-9/10,16-DHFA), the predominant monomer of the Arabidopsis flower cutin, was significantly different between WT and *ga1-3* and between *ga1-3* and the *penta* mutant (Figure S9). Nevertheless, SEM observation did not reveal any significant changes in the petal surface of the open flowers in the mutant plants (Figure S9). Since we applied exogenous GA to *ga1-3* plants to induce flowering [43] prior to the SEM observation, this might explain the absence of a surface phenotype in mutant petal surface. All together, these results suggest that *SHN* transcription factors might play a key role in the GA-mediated flower organ development regulatory network.

## Discussion

Aerial plant organs display tremendous variation in their surface topography and composition of the cuticular layer covering their outer epidermis. This diversity in the exterior layer is essential for both organogenesis and the interaction with the environment. In flowers for instance, the typical surface of organs is vital for their function as it ensures their proper development by preventing postgenital fusions while at the same time mediating the interaction with insect pollinators [44]–[45]. Whereas many molecular components of pathways determining flower organ initiation and identity have been characterized to date [46], our knowledge regarding formation and function of their outer surface, namely the cuticle, is limited. Here, in-depth analysis of Arabidopsis plants in which the three SHN transcription factors were co-silenced revealed that these regulators play a prominent role in patterning floral organ surface by controlling metabolism of cuticular lipids and possibly the associated cell wall components.

### SHINE transcription factors act redundantly to ensure proper floral organ morphology and surface formation

The lack of any visual phenotype in floral organs of *SHN1/WIN1* silenced plants ([18]; Figure S3), pointed to functional redundancy among the 3 SHN clade members. Even though expression of either one of the three *SHN* genes was not entirely reduced, the use of an artificial microRNA targeting the entire clade was sufficient to obtain several, striking, visual phenotypes that matched the previously described *SHN* genes expression patterns [16]. Floral organs were affected, likely as a result of altered cuticle composition, structure and consequently permeability. However, cuticle alteration might not be the only explanation to the defects observed in organ formation since they might also be a result of *SHN* genes effect on the process of epidermal cell differentiation and development. This was evidenced in the altered epidermal cells size and shape in petals and sepals of the 35S*:miR-SHN1/2/3* plants. These strong epidermis phenotypes (in pavement cells, trichomes and stomata) observed previously in plants overexpressing either one of the three *SHN* genes support this proposal [16].

Down regulation of the *SHN* clade genes had an additional effect on floral organs as SEM and transmission electron microscopy (TEM) revealed changes in nanoridges that typically decorate surfaces of flower organs [44]. Formation of nanoridges in Arabidopsis flowers was recently associated with cutin, particularly with C_16_-9/10,16-DHFA, the major monomer of Arabidopsis petal cutin [22]–[23], that was also dramatically reduced in the 35S:miR-*SHN1/2/3* plants. However, the absence of nanoridges on the surface of tomato fruit that also contains C_16_-9/10,16-DHFA as a major monomer, suggests additional factors including polymer structure and distribution that mediate nanoridge formation [23], [47].

### SHINE transcription factors mediate floral organ adhesion and separation

Earlier work using promoter-reporter assays suggested that SHN transcription factors act not only in the interface between the plant and its environment but also at the interface between cells and cell layers [16]. Of particular interest was *SHN2* that showed strict expression in the anther and silique dehiscence zones upon organ maturation. The proposed role of SHN transcription factors in the adhesion of cell layers was strongly corroborated by the recent finding that an *SHN*-like gene in barley (*Nud*) mediates the contact of the caryopsis surface to the inner side of the hull by forming a specialized lipid layer [19]. In this study we detected earlier abscission of floral organs in the silenced lines which corresponded well with *SHN* genes expression in the base of sepals, petals, stamens and siliques in the abscission region. Organ separation events including pod shatter, seed detachment from the maternal plant, pollen separation after meiosis, anther dehiscence and floral organ abscission, are thought to be associated with alterations to properties of the cell wall matrix, mainly pectins and wall proteins [1], [48]–[49]. The pectin degradation activity of polygalacturonases (PGs) has been linked with all separation events described above. Recently, three Arabidopsis PGs have been associated with cell separation during reproductive development [50]. One of these, *ADPG1*, displayed altered expression in the 35S*:miR-SHN1/2/3* plants and its promoter was shown here to be activated by SHN1/WIN1 and SHN2. Thus, SHN action on organ adhesion/separation possibly combines modification to cuticular lipids (i.e. cutin) as well as pectins of the cell wall.

### Cutin, cell wall, and possibly suberin-associated genes are downstream targets of SHINE transcription factors

Array analysis revealed a concise set of genes that are putative downstream targets of the SHN transcription factors in flower buds, only two out of them (*CYP86A4* and *CYP86A7*) overlapped with the previously reported group of 11 SHN1/WIN1 putative targets [18]. This could be explained by the fact that while Kannagara et al. (2007) detected genes that were upregulated following induction of *SHN1/WIN1* in fully expanded leaves [18], we examined flower buds in which the *SHN* clade genes were co-silenced. Thus, genes from these two experiments most likely represent downstream targets in either leaves or flowers or both tissues. Together, these studies also demonstrated that wax load changes in the *SHN* overexpression lines were probably an indirect effect.

SHN transcription factors emerge as regulators of genes derived from four prominent families associated with the cuticle including two cytochrome P450s of the CYP86A clade (*CYP86A4* and *CYP86A7*), *BDG3*, encoding one of the five BDG1-like proteins [24], three genes of the large family of GDSL-motif lipase/hydrolases [39] and one of the eight-member clade of fatty acyl-CoA reductases [25]. Apart from the latter, these genes or their family members have been reported to be involved in either cutin biosynthesis or polymer assembly in the extracellular matrix in plant reproductive organs [10], [23], [51]–[53]. FAR1 has been recently associated with formation of suberin, a polymer that is structurally related to cutin and is often deposited following cell to cell separation in aerial organs to form a protection layer that will shield against penetration of pathogens and dehiscence [25], [54]. Below ground, endodermal suberin is thought to regulate the apoplastic movement of water and solutes into the stele [55]–[56]. The *SHN3* expression in roots ([16], Figure S10) and the endodermal expression of *FAR1*, *BDG3*, *CYP86A4* and *At1g16760* (Figure S10) suggested that the latter 4 genes are targets of SHN transcription factors both above and below ground. Hence, SHN transcription factors and their targets are not only involved in cutin assembly in reproductive organs but are likely to play a role in root suberin deposition. CYP86A4 was suggested to provide ω-hydroxylation activity that is complementary to CYP86A1 in the biosynthesis of suberin [57] and FAR1 was recently reported to be associated with generating primary fatty alcohols for suberin deposition [25]. However, the role of *BDG3* and *At1g16760* in root suberin remains to be determined.

### SHINE transcription factors and the GA-mediated flower development network

Previous reports regarding the SHN clade members highlighted their role in regulating the biosynthesis of cuticular lipids for surface formation [16]–[18]. However, the results of the present study imply that activity of these factors goes beyond regulating a single metabolic pathway (i.e. cutin) for cuticle formation and they take part in the genetic program that mediates floral organ morphogenesis, more specifically in determining organ size and shape as well as the formation of specialized epidermis cell types (e.g. the petal conical cells). Related to this, gene expression changes detected in the 35S:*miR:SHN1/2/3* flower buds strikingly resemble the ones implicated in the formation of the single epidermis cotton fiber cell during its elongation. These include altered expression of genes associated with cell wall loosening through modification of pectin [58], genes associated with the build-up of a higher turgor by increased accumulation of the major osmoticum such as soluble sugars, K^+^, and malate [27], redox-related genes [59]–[60], genes related to phytohormone biosynthesis and signaling cascades [61].

Flowering in Arabidopsis consists of three distinct phases: floral initiation, floral organ initiation and floral organ growth. Earlier studies on GA signaling revealed that GA promotes Arabidopsis petal, stamen, and anther development by opposing the function of the DELLA proteins [62] and that GA signaling is not required for floral organ specification but essential for the normal growth and development of these organs [63]. Different combinations of DELLA proteins are key to floral organ development (RGA, RGL1, RGL2), because individual DELLA proteins have different temporal and spatial expression patterns [62]. The unique temporal and spatial expression patterns of *SHN* clade genes in the flower tissues [16] and their distinct expression patterns in response to the alteration of the GA signaling reported here suggest that SHNs might be part of GA floral regulatory networks. In this context, GA might act as a positive regulator of *SHN1/WIN1* in the regulation of floral organs development (i.e. elongation of petal, stamen, and anther) [37], [62] in the early stages of flower development. In addition, GA emerges as a negative regulator of *SHN2* in modulating the cell separation processes related to silique and anther dehiscence, floral organ abscission in the later stages of flower development. Hence, GA might be involved in cuticle assembly during the expansion of petals and other floral organs. The growth and elongation of organs requires the interaction between the outer and inner cell layers, which is coordinated by hormonal signals [4]–[5]. GA has been shown to promote cutin synthesis during other growth related processes including the rapidly growing internodes of deep-water rice [64], in extending stems of peas [65], and in developing tomato fruit [66]. Similarly, in this study, GA application resulted in a significant increase in the cutin load of *ga1-3* mutant flowers. Future studies positioning the SHN proteins in the wide genetic network that controls flower development will shed light on how cuticle and cell wall metabolism is coordinated with the processes of flowering and fertility.

## Materials and Methods

### Plant material

All *Arabidopsis* plants used in *miR*-*SHN1/2/3* experiment were in the Col-0 genetic background, while those used for DELLA or GA experiment were in Ler genetic background. Plants were grown on a soil mixture in a growth room at 20°C, 70% relative humidity, a 16/8-h light/dark cycle at a fluorescent light intensity of 100 µmol m^−2^s^−1^. All knock out lines were bought from either ABRC or NASC, while GA biosynthesis and signaling mutant were kind gifts from Hao Yu (National University of Singapore, Singapore) and David Weiss (The Hebrew University, Israel). Exogenous GA application was carried out as described [67] with minor modifications. 100 mM GA_3_ or ethanol containing water was fine sprayed daily for 6 days on 6-week-old plants, and the buds were collected for analysis.

### Generation of transgenic plants

For the 35S:*miR-SHN1/2/3* construct, the designed artificial *miR-SHN1/2/3* sequence was directly synthesized from BIO S&T (Bio S&T Inc., Montreal, Canada). After being sequenced, it was put into pART7 vector, and finally subcloned to pART27. Transformation to *Agrobacterium tumefaciens* strain GV3101 was done via electroporation and planta transformation was done via floral dipping as described [68]. Promoter sequences of the putative SHN target genes (approximately 2 kb upstream of the start codon) were cloned from WT genomic DNA, and coding sequences of the three members of SHN clade were cloned from WT flower cDNA, using yellow Taq DNA polymerase (Roboklon Gmbh, Berlin, Germany) with corresponding gene specific primer pairs (Table S1). Those promoters and TFs were cloned into pGreen II 0800-LUC vector and pBIN plus vector, respectively, and then transformed to *Agrobacterium tumefaciens* strain GV3101. All DNA sequence cloned were examined by direct sequencing.

### Histological observations

Toluidine blue examination of cuticle permeability was performed as previously described [69]. For Rethinium red staining, the inflorescences of 7-week-old plants were fixed and embedded in LR White resin (London Resin Co., Basingstoke, UK) as described previously [70]. Sections were cut to a thickness of 0.5–1 mm using a diamond knife on an Ultracut microtome (Leica) and sections were collected on glass slides. The slides were stained with 0.1% Rethinium red for 5 min and washed with double distilled water, and then observed with Nikon ECLIPSE E800 microscope.

### Electron microscopy

All electron microscopy works were done as previously described [22]. For scanning electron microscopy (SEM), flowers from 7-week-old plants were collected, fixed with glutaraldehyde using standard SEM protocol [71], dried using critical point drying (CPD), mounted on aluminum stubs and sputter-coated with gold. SEM was performed using an XL30 ESEM FEG microscope (FEI) at 5–10 kV. For TEM, flowers from 7-week-old plants were collected and processed using a standard protocol [72]. The Epon-embedded samples were sectioned (70 nm) using an ultramicrotome (Leica) and observed with a Technai T12 transmission electron microscope (FEI).

### RNA extraction and microarray analysis

Total RNA was extracted from closed buds from 7-weeks-old WT and homozygous 35S*:miRSHN1/2/3* T3 plants using RNeasy Plant Mini Kit (Qiagen) with an on column DNAse treatment. The subsequent microarray analysis and qRT-PCR analysis were performed as described previously [21]. For microarray analysis, the double-stranded cDNA was purified and served as a template in the subsequent in-vitro transcription reaction for complementary RNA (cRNA) amplification and biotin labeling. The biotinylated cRNA was cleaned, fragmented and hybridized to Affymetrix ATH1 Genome Array chips. Statistical analysis of microarray data was performed using the Partek® Genomics Suite (Partek Inc., St. Louis, Missouri) software. CEL files (containing raw expression measurements) were imported to Partek GS. The data was preprocessed and normalized using the RMA (Robust Multichip Average) algorithm [73]. The normalized data was processed by PCA (Principal Component Analysis) and hierarchical clustering to detect batch or other random effects. To identify differentially expressed genes one-way ANOVA analysis of variance was applied. Gene lists were created by filtering the genes based on: fold change, p<0.01, and signal above background in at least one microarray. Up-regulated genes were defined as those having a greater than or at least 1.5-fold linear intensity ratio while down-regulated genes were defined as those having a less than or at most −1.5-fold linear intensity ratio. The experiment was performed in duplicate, preparing two independent biological replicates from 5–6 plants each.

### Wax and cutin analysis

Waxes were extracted and analyzed as described [22]. For cutin analysis, soluble lipids were extracted from leaf and closed buds by dipping them in 10 ml of a methanol/chloroform (1∶1, v/v) mixture for 14 days (solvent changed daily). The tissues were dried, weighed (about 10–20 mg) and kept in N_2_ till analysis. The cutin was depolymerized and analyzed as described previously [22], [54].

### Fourier transform infrared (FTIR) spectroscopy

Petals from 7-week-old flowers were collected (60 petals each sample, n = 8), cleared with chloroform and methanol (1∶1), and then air-dried overnight [74]. Samples were ground with solid crystalline KBr to fine powder and pressed to 1-mm tablelets. FTIR spectra were acquired in the absorbance mode at a resolution of 4 cm^−1^ with 32 co-added scans at wave number range 4000 to 250 cm^−1^ using a NICOLE1 380 FITR Spectrometer (Thermo Electron Corporation). Each spectrum was baseline corrected and spectral area normalized prior to generating average spectra and digital subtraction spectra. Primary component analysis was performed using Multiple Experiment Viewer.

### Transverse section preparation and immunocytochemistry

Inflorescence stems transverse sections were prepared according to Willats et al [75]. Regions (0.5 cm long) of 7-week-old Arabidopsis stem (3^th^ internodes from the bottom) were excised and sectioned by hand to a thickness of ∼100–300 µm. Sections were immediately placed in fixative consisting of 4% paraformaldehyde in 50 mM PIPES, 5 mM MgSO_4_, and 5 mM EGTA. Following 30 min of fixation, sections were washed in the PIPES buffer, and then in 1× PBS buffer. Petals and gynoecium transverse section were prepared as described [65] and *In vitro* immunocytochemistry was carried out as described by Verhertbruggen et al [34]. Sections were incubated for 1.5 h in 5-fold dilution of two new rat monoclonal antibody hybridoma supernatant (LM19 and LM20) diluted in 5% Milk/PBS, respectively. After being washed by gently rocking in PBS at least three times, sections were incubated with a 100-fold dilution of anti-rat IgG (whole molecule) linked to fluorescein isothiocyanate (FITC) in 5% Milk/PBS for 1.5 h in darkness. After washing in PBS for at least 3 times, sections were mounted in a glycerol∶PBS (vol∶vol, 1∶1) solution. Immunofluorescence was observed with Nikon ECLIPSE E800 microscope equipped with epifluorescence irradiation and DIC optics. Images were captured with a camera and NIS-Elements BR30 software.

### Dual luciferase assay

Transient assay was carried out as described [40] with the exception that 150 µg/ml instead of acetosyringone was included in the infiltration media [76]. Luminescence was measured using Modulus Microplate Luminometer (Turner Biosystems, Sunnyvale, CA) by mixing 20 µl sample extract with 80 µl Luciferase assay reagent or Renillase assay reagent, respectively, and the data was collected as ratio. Background controls were run with only the transcription factor, promoter-LUC, and pBIN Plus empty vector, and pBIN Plus empty vector with promoter-LUC in the preliminary assay, and pBIN Plus empty vector with promoter-LUC was chosen later for background control in all experiments due to its relatively higher induction of Luciferase activity than other plasmid tested.

## Supporting Information

Figure S1 Overexpression of the *miR-SHN1/2/3* cleaves the targeted *SHN* genes and causes morphological changes in reproductive organs. (A) Predicted folding and dicing of the pre *miR164a* backbone before (left) and after (right) replacement of *miR164* with *miR-SHN1/2/3* sequence. *miR164a* (left panel) or *miR-SHN1/2/3* (right panel) sequence is red colored. (B) RLM-RACE detection of cleaved products of the three *SHN* transcripts in 35S*:miR-SHN1/2/3* plants but not WT plants (Left panel). M, marker; 4, 21, and 24, 3 independent 35S*:miR-SHN1/2/3* T2 lines. (C) Sequence alignment of the *miR-SHN1/2/3* binding sites and summary of cleavage analysis by direct sequencing of RLM-RACE products in Arabidopsis. *SHN1/WIN1* (*At1g15360*), *SHN2* (*At5g11190*), and *SHN3(At5g25390)*. Mismatches are marked red and cleavage site is indicated by arrow. DS, direct sequencing. (D–E) 2-week-old seedlings. (F–G) Toluidine Blue (TB) stained 4-week-old seedlings. (H–I) TB stained inflorescences. Arrows point to the stained region. (J–K) 6-week-old inflorescences. Arrows point to the floral organ abscission position. (L–M) SEM images of a folded carpel and a twisted petal, respectively, derived from 35S*:miR-SHN1/2/3* flower. (N–O) TEM images of the sepal surfaces. Note the changes in the shape of epidermal cells (ec). (P–Q) TEM images of the filament surfaces. Scale bars: L and M, 100 µm; N, 0.9 µm; O and P, 1 µm; Q, 4 µm.
(0.71 MB PDF)

Figure S2 Defective nanoridge phenotypes observed on the surfaces of floral organs other than petals in 35S*:miR-SHN1/2/3* plants by SEM. (A–B) Adaxial sepal surfaces. (C–D) Abaxial sepal surfaces. (E–F) Filament surfaces. (G–H) Pedicle surfaces. (I–J) Nectary surfaces. (K–L) Style surfaces. Note the disappearance or reduction of the deposition of nanoridges on the surfaces of those floral organs in 35S*:miR-SHN1/2/3*.
(0.90 MB PDF)

Figure S3 *SHN1/WIN1* silencing does not affect floral organ morphology and surface characteristics. (A) Inflorescence of *SHN1/WIN1* RNAi (*SHN1/WIN1* R) appears the same as that of WT. (B) A closer view shows no morphological difference between WT and *SHN1/WIN1* R line inflorescence. (C) Floral bud morphology in WT and *SHN1/WIN1* R line is similar. (D) Flowers of WT and *SHN1/WIN1* R line are similar. (E–F) SEM images of the adaxial petal surface displays no changes in the patterning of the cuticular ridges between WT (E) and the *SHN1/WIN1* R plants (F). (G–H) SEM images of the abaxial petal indicate no changes in the patterning of the cuticular ridges in the *SHN1/WIN1* R plants (H) as compared with WT (G).
(0.27 MB PDF)

Figure S4 Real time RT-PCR validation of the expression of differential expressed genes revealed by microarray analysis in flower buds. Values present means and standard errors (n = 3). *, p<0.05; **, p<0.01. White bars, WT; Gray bars, 35S*:miR-SHN1/2/3*.
(0.04 MB PDF)

Figure S5 Profiling of leaf cutin, leaf and flower waxes, and bud cell walls and seed mucilage monosaccharides. (A) Cutin profiling of mature rosette leaves. FA, fatty acids; DFA, α,ω-dicarboxylic FA; 2-HFA, 2-hydroxy fatty acids; ω-HFA, ω-hydroxy fatty acids; HDFA, hydroxy dioic aicds. Values represent means and standard errors (n = 3). (B–C) Wax profiling of mature rosette leaves (B) and flowers (C), respectively. Inserted is the total leaf wax. ALC, alcohols; ALD, aldehydes; ALK, alkanes; FA, fatty acids, KET, ketones. Values represent means and standard errors (n = 4). *, p<0.05. (D–E) Monosaccharide compositions of bud cell walls (D) and seed mucilage (E). Values represent the means and SE (bud: n = 5; seed mucilage: n = 4). Xyl: xylose; Ara: arabinose; Rha: rhamnose; Fuc: fucose; Gal: galactose; Man: mannose; GalA: galacturonic acid.
(0.31 MB PDF)

Figure S6 *In silico* coexpression analysis. (A) Network of *SHN1/WIN1* co-expressed genes as revealed by ATTED-II from Tair: http://atted.jp/; Red and green shaded genes represent up-and down-regulated genes in 35S:miR-SHN1/2/3 buds, respectively. (B) Co-correlation scatter plot (2-D Pearson Correlation Coefficients) of some *SHN* target genes with *SHN1* and *SHN3*, respectively, generated using Arabidopsis Coexpression Data Mining Tools (http://www.arabidopsis.leeds.ac.uk/act).
(0.28 MB PDF)

Figure S7 Genetic modifying putative target genes of SHINE alters petal surface pattering. (A–B) SEM images of the WT petal adaxial and abaxial side, respectively. (C–P) SEM images of petal epidermis (adaxial and abaxial, respectively) derided from knock out plants of *At5g23970* (C–D); *bdg3* (E–F), *At5g03350* (G–H), *cyp86a4-1* (I–J), *cyp86a4-2* (K–L), *at5g33370-1*(M–N), *at5g33370-2* (O–P). (Q–T) SEM images of petal epidermis (adaxial and abaxial, respectively) derived from artificial microRNA co-silenced *CYP86A* and *GDSL-lipase* plants (Q–R, 35S*:miR-CYP84A4/A7*; S–T, 35S*:miR-At5g33370/At3g04290*. *At3g04290/LTL1* is the closest GDSL lipase to *At5g33370* in the same family). (U) Real time RTPCR analysis validation of the downregulation of the expression of both *CYP86A4* and *CYP86A7* in 35S:*miR-CYP86A4/7* plants. (V–W) RT-PCR confirmation of the activation of the microRNA machinery showing the expression of microRNA precursor in various transgenic plant lines. (X) The T-DNA insertion positions of these mutants mentioned above.
(0.91 MB PDF)

Figure S8 GA regulates the expression of *SHN1/WIN1* and several SHINE putative target genes in a DELLA-dependent manner. (A) GA up-regulated *SHN1/WIN1* and 13 SHN putative target genes in a DELLA dependent way in the young flower buds. Top panel: GA up-regulated (WT vs. *ga1-3*); Bottom panel: DELLA up-regulated (*penta* vs. *ga1-3*). (B) GA down-regulated 4 SHN putative target genes in a DELLA dependent way in the young flower buds. Top panel: GA down-regulated (WT vs. *ga1-3*); Bottom panel: DELLA down-regulated (*penta* vs. *ga1-3*). All data were adopted from Cao et al., 2006 [5]. Values are means and standard errors (n = 6). *ga1-3*, loss of function mutant in the GA1 gene which encodes an enzyme involved in GA biosynthesis; *penta*, GA-deficient quadruple mutant *ga1-3 gai-t6 rga-t2 rgl1-1 rgl2-1*; In *ga1-3*, all DELLA proteins are active.
(0.18 MB PDF)

Figure S9 Petal Surface Morphology and Profiling of flower cutin and waxes in GA and or DELLA mutants. (A–D) SEM images of the petal surfaces. A and C, WT adaxial and abaxial petal surface, respectively; B and D, *ga1-3* adaxial and abaxial petal surface, respectively. (E) Cutin profiling of open flowers (Inserted is the total cutin). FA, fatty acids; DFA, α,ω-dicarboxylic FA; ω-HFA, ω-hydroxy FA; DHFA, C16/9,10-HFA; HDFA, C16-9/10-hydroxy DFA; 2-HFA, 2-hydroxy FA. Values represent means and standard errors (n = 4). Different letters indicate the significant difference (p<0.05). (F) Wax profiling of open flowers (Inserted is the total wax). FA, fatty acids; ALC, alcohols; ALK, alkanes; BR ALK, branched alkanes; KET, ketones. Values represent means and standard errors (n = 4).
(0.66 MB PDF)

Figure S10 Gus expression pattern of SHN3 in the roots and mRNA levels of four SHINE putative target genes in translatomes of different cell populations of Arabidopsis. (A) Gus staining of SHN3 observed in the central cylinder of primary and lateral roots.(B) Cross section through a primary root (maturation zone) showing GUS staining of SHN3 in the parenchymatic cells of the stele. (C–F) Absolute signal values of four putative SHN/WIN target gene transcripts in translatomes isolated from cell populations visualized via the eFP platform (efp.ucr.edu/).
(0.25 MB PDF)

Table S1 List of primers used in this study.
(0.03 MB XLS)

Table S2 List of genes co-expressed with SHN1/WIN1 or SHN3.
(6.98 MB XLS)

Text S1 Supporting Materials and Methods and References.
(0.04 MB DOC)
