# Supplementary material for: SHINE Transcription Factors Act Redundantly to Pattern the Archetypal Surface of Arabidopsis Flower Organs
Source: PLoS Genet. 2011 May 26;7(5):e1001388. doi: 10.1371/journal.pgen.1001388 (PMC3102738; doi:10.1371/journal.pgen.1001388)
Supplement: Figure S1 — Overexpression of the miR-SHN1/2/3 cleaves the targeted SHN genes and causes morphological changes in reproductive organs. (A) Predicted folding and dicing of the pre miR164a backbone before (left) and after (right) replacement of miR164 with miR-SHN1/2/3 sequence. miR164a (left panel) or miR-SHN1/2/3 (right panel) sequence is red colored. (B) RLM-RACE detection of cleaved products of the three SHN transcripts in 35S:miR-SHN1/2/3 plants but not WT plants (Left panel). M, marker; 4, 21, and 24, 3 independent 35S:miR-SHN1/2/3 T2 lines. (C) Sequence alignment of the miR-SHN1/2/3 binding sites and summary of cleavage analysis by direct sequencing of RLM-RACE products in Arabidopsis. SHN1/WIN1 (At1g15360), SHN2 (At5g11190), and SHN3(At5g25390). Mismatches are marked red and cleavage site is indicated by arrow. DS, direct sequencing. (D–E) 2-week-old seedlings. (F–G) Toluidine Blue (TB) stained 4-week-old seedlings. (H–I) TB stained inflorescences. Arrows point to the stained region. (J–K) 6-week-old inflorescences. Arrows point to the floral organ abscission position. (L–M) SEM images of a folded carpel and a twisted petal, respectively, derived from 35S:miR-SHN1/2/3 flower. (N–O) TEM images of the sepal surfaces. Note the changes in the shape of epidermal cells (ec). (P–Q) TEM images of the filament surfaces. Scale bars: L and M, 100 µm; N, 0.9 µm; O and P, 1 µm; Q, 4 µm. (0.71 MB PDF) [file pgen.1001388.s001.pdf]

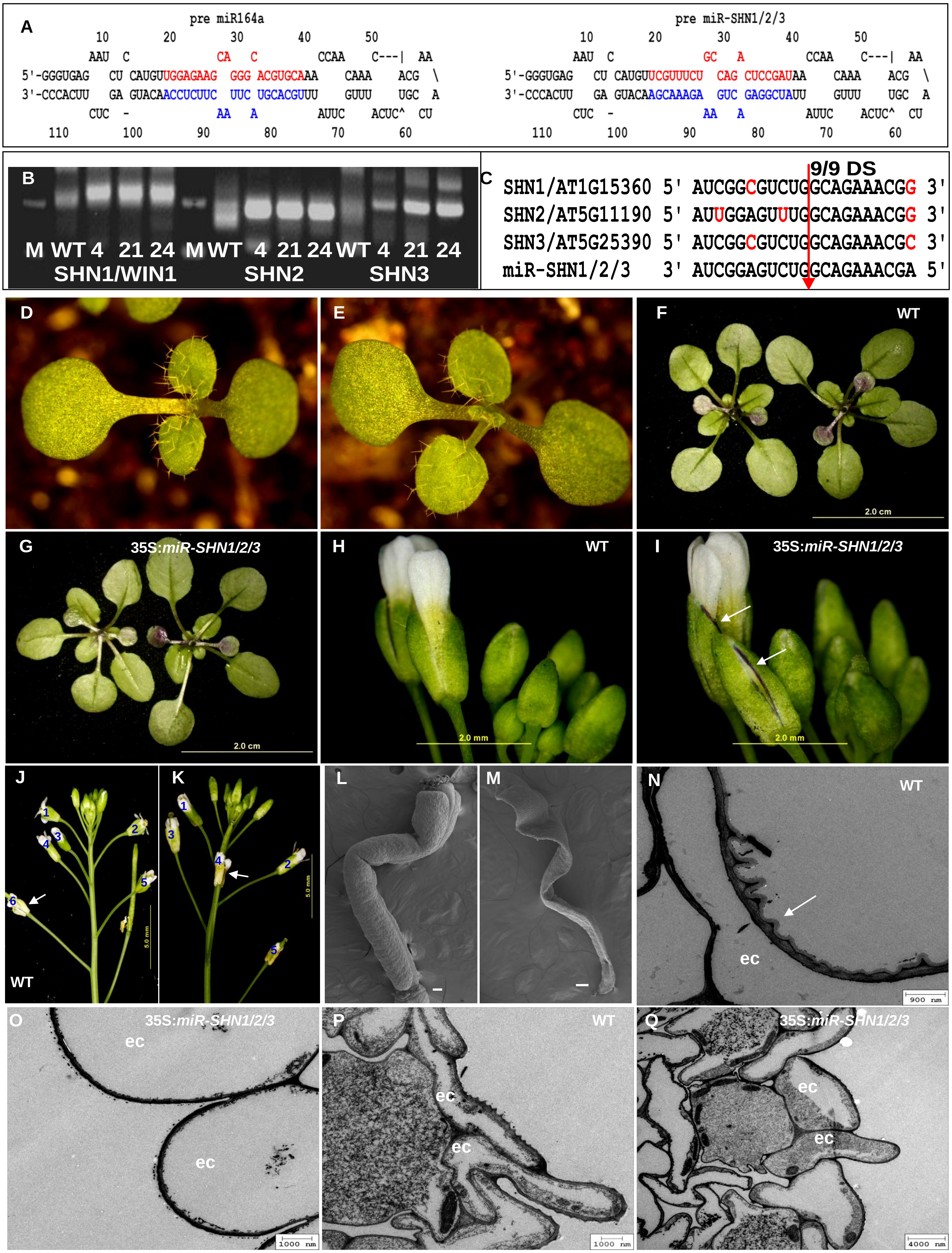

Shi et al., Figure S1

Figure S1. **Overexpression of the *miR-SHN1/2/3* cleaves the targeted *SHN* genes and causes morphological changes in reproductive organs**. (A) Predicted folding and dicing of the pre *miR164a* backbone before (left) and after (right) replacement of *miR164* with *miR-SHN1/2/3* sequence. *miR164a* (left panel) or *miR-SHN1/2/3* (right panel) sequence is red colored. (B) Cleaved products of the three *SHN* transcripts were detected by RLM-RACE in 35S*:miR-SHN1/2/3* plants but not in WT plants (Left panel). M, marker; 4, 21, and 24, 3 independent 35S*:miR-SHN1/2/3* T2 lines. (C) Sequence alignment of the *miR-SHN1/2/3* binding sites and summary of cleavage analysis by direct sequencing of RLM-RACE products in Arabidopsis. *SHN1/WIN1* (*At1g15360*), *SHN2* (*At5g11190*), and *SHN3(At5g25390).* Mismatches are marked red and cleavage site is indicated by arrow. DS, direct sequencing. (D-E) 2-week-old seedlings. (F-G) Toluidine Blue (TB) stained 4-week-old seedlings. (H-I) TB stained inflorescences. Arrows point to the stained region. (J-K) 6-week-old inflorescences. Arrows point to the floral organ abscission position. (L-M) SEM images of a folded carpel and a twisted petal, respectively, derived from 35S*:miR-SHN1/2/3* flower. (N-O) TEM images of the sepal surfaces. Note the changes in the shape of epidermal cells (ec). (P-Q) TEM images of the filament surfaces. Scale bars: L and M, 100 μm; N, 0.9 μm; O and P, 1 μm; Q, 4 μm.
