# Supplementary material for: SHINE Transcription Factors Act Redundantly to Pattern the Archetypal Surface of Arabidopsis Flower Organs
Source: PLoS Genet. 2011 May 26;7(5):e1001388. doi: 10.1371/journal.pgen.1001388 (PMC3102738; doi:10.1371/journal.pgen.1001388)
Supplement: Figure S2 — Defective nanoridge phenotypes observed on the surfaces of floral organs other than petals in 35S:miR-SHN1/2/3 plants by SEM. (A–B) Adaxial sepal surfaces. (C–D) Abaxial sepal surfaces. (E–F) Filament surfaces. (G–H) Pedicle surfaces. (I–J) Nectary surfaces. (K–L) Style surfaces. Note the disappearance or reduction of the deposition of nanoridges on the surfaces of those floral organs in 35S:miR-SHN1/2/3. (0.90 MB PDF) [file pgen.1001388.s002.pdf]

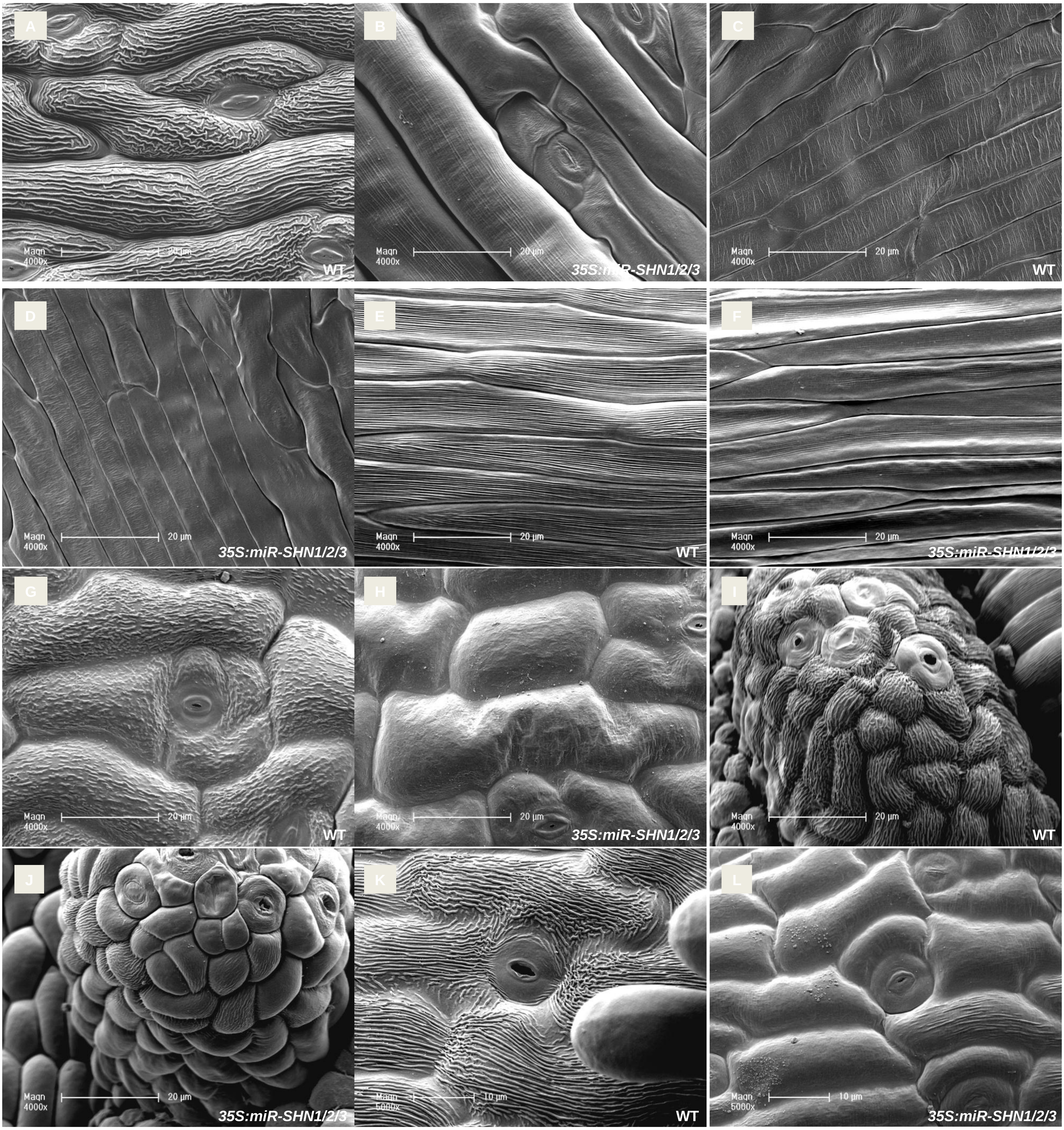

Figure S2. Defective nanoridge phenotypes observed on the surfaces of floral organs other than petals in 35S*:miR-SHN1/2/3* plants by SEM. (A-B) Adaxial sepal surfaces. (C-D) Abaxial sepal surfaces. (E-F) Filament surfaces. (G-H) Pedicle surfaces. (I-J) Nectary surfaces. (K-L) Style surfaces. Note the disappearance or reduction of the deposition of nanoridges on the surfaces of those floral organs in 35S*:miR-SHN1/2/3*.
