# Supplementary material for: SHINE Transcription Factors Act Redundantly to Pattern the Archetypal Surface of Arabidopsis Flower Organs
Source: PLoS Genet. 2011 May 26;7(5):e1001388. doi: 10.1371/journal.pgen.1001388 (PMC3102738; doi:10.1371/journal.pgen.1001388)
Supplement: Figure S3 — SHN1/WIN1 silencing does not affect floral organ morphology and surface characteristics. (A) Inflorescence of SHN1/WIN1 RNAi (SHN1/WIN1 R) appears the same as that of WT. (B) A closer view shows no morphological difference between WT and SHN1/WIN1 R line inflorescence. (C) Floral bud morphology in WT and SHN1/WIN1 R line is similar. (D) Flowers of WT and SHN1/WIN1 R line are similar. (E–F) SEM images of the adaxial petal surface displays no changes in the patterning of the cuticular ridges between WT (E) and the SHN1/WIN1 R plants (F). (G–H) SEM images of the abaxial petal indicate no changes in the patterning of the cuticular ridges in the SHN1/WIN1 R plants (H) as compared with WT (G). (0.27 MB PDF) [file pgen.1001388.s003.pdf]

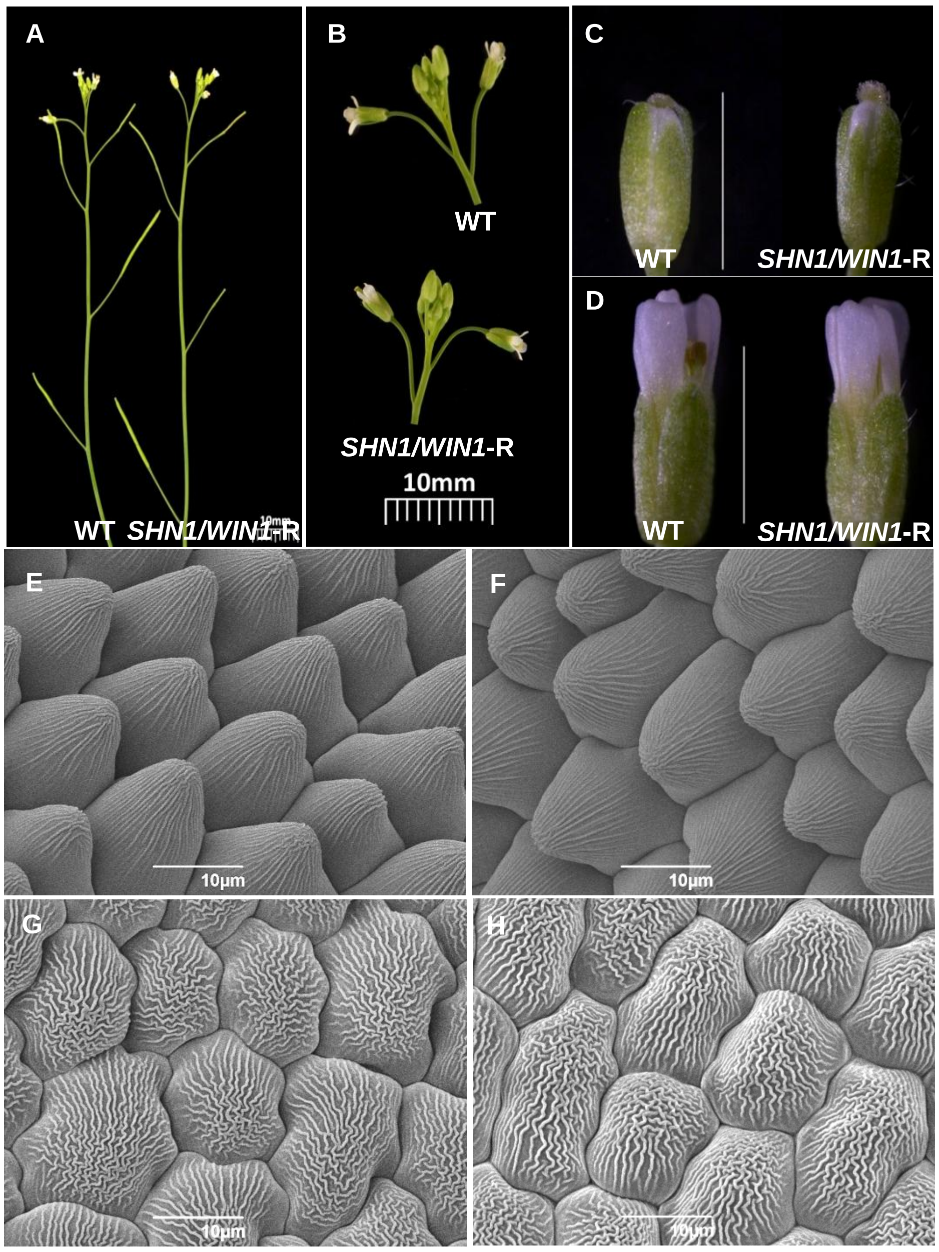

Figure S3. *SHN1/WIN1* silencing does not affect floral organ morphology and surface characteristics. (A) Inflorescence of *SHN1/WIN1* RNAi (*SHN1/WIN1* R) appears the same as that of WT. (B) A closer view shows no morphological difference between WT and *SHN1/WIN1* R line inflorescence. (C) Floral bud morphology in WT and *SHN1/WIN1* R line is similar. (D) Flowers of WT and *SHN1/WIN1* R line are similar. (E-F) SEM images of the adaxial petal surface display no changes in the patterning of the cuticular ridges between WT (E) and the *SHN1/WIN1* R plants (F). (G-H) SEM images of the abaxial petal indicate no changes in the patterning of the cuticular ridges in the *SHN1/WIN1* R plants (H) as compared with WT (G).
