# Supplementary material for: SHINE Transcription Factors Act Redundantly to Pattern the Archetypal Surface of Arabidopsis Flower Organs
Source: PLoS Genet. 2011 May 26;7(5):e1001388. doi: 10.1371/journal.pgen.1001388 (PMC3102738; doi:10.1371/journal.pgen.1001388)
Supplement: Figure S4 — Real time RT-PCR validation of the expression of differential expressed genes revealed by microarray analysis in flower buds. Values present means and standard errors (n = 3). *, p<0.05; **, p<0.01. White bars, WT; Gray bars, 35S:miR-SHN1/2/3. (0.04 MB PDF) [file pgen.1001388.s004.pdf]

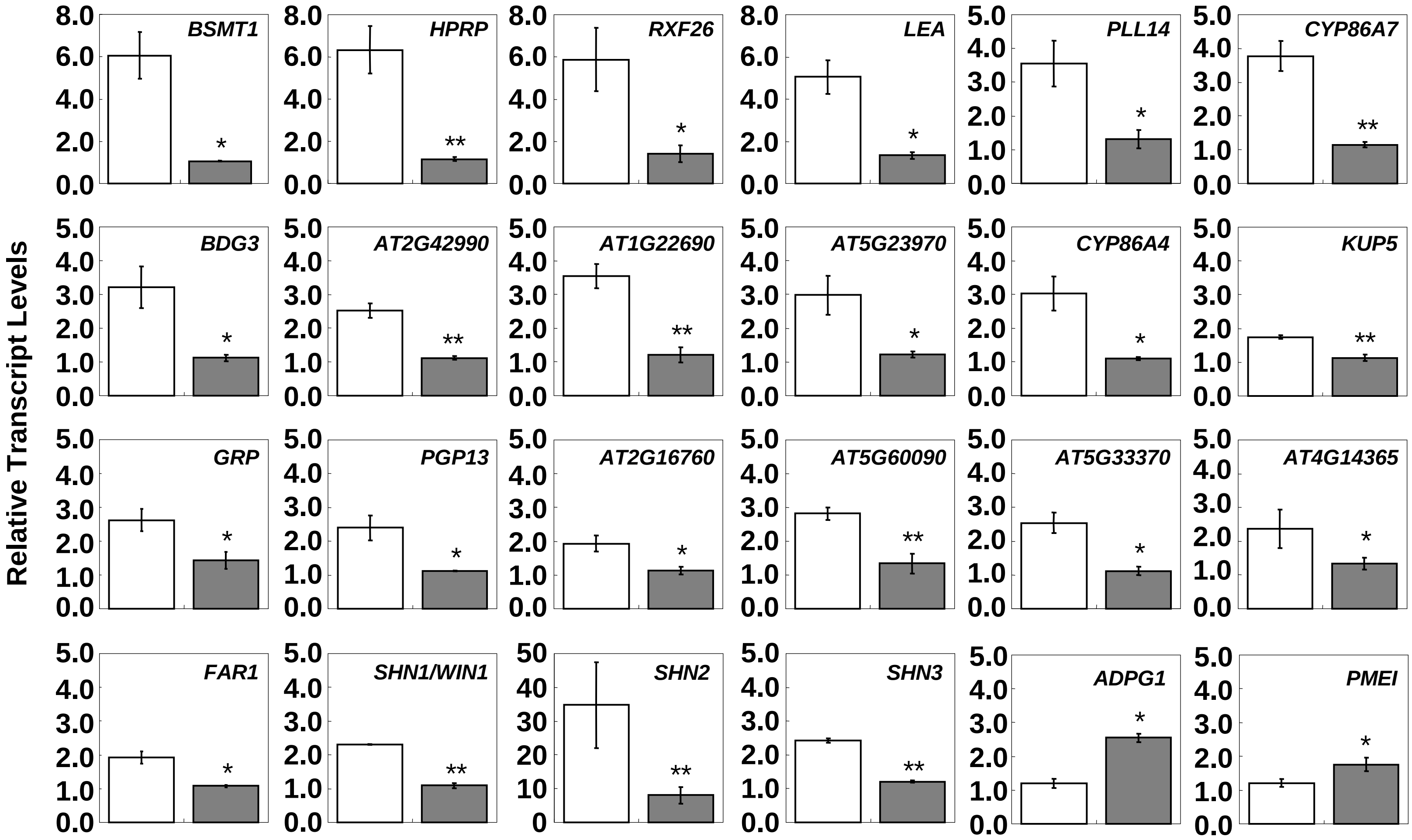

Figure S4. Real time RT-PCR validation of the expression of differential expressed genes revealed by microarray analysis in flower buds. Values present means and standard errors (n=3). *, p<0.05; **, p<0.01. White bars, WT; Gray bars, 35S:*miR-SHN1/2/3*.
