# Supplementary material for: SHINE Transcription Factors Act Redundantly to Pattern the Archetypal Surface of Arabidopsis Flower Organs
Source: PLoS Genet. 2011 May 26;7(5):e1001388. doi: 10.1371/journal.pgen.1001388 (PMC3102738; doi:10.1371/journal.pgen.1001388)
Supplement: Figure S5 — Profiling of leaf cutin, leaf and flower waxes, and bud cell walls and seed mucilage monosaccharides. (A) Cutin profiling of mature rosette leaves. FA, fatty acids; DFA, α,ω-dicarboxylic FA; 2-HFA, 2-hydroxy fatty acids; ω-HFA, ω-hydroxy fatty acids; HDFA, hydroxy dioic aicds. Values represent means and standard errors (n = 3). (B–C) Wax profiling of mature rosette leaves (B) and flowers (C), respectively. Inserted is the total leaf wax. ALC, alcohols; ALD, aldehydes; ALK, alkanes; FA, fatty acids, KET, ketones. Values represent means and standard errors (n = 4). *, p<0.05. (D–E) Monosaccharide compositions of bud cell walls (D) and seed mucilage (E). Values represent the means and SE (bud: n = 5; seed mucilage: n = 4). Xyl: xylose; Ara: arabinose; Rha: rhamnose; Fuc: fucose; Gal: galactose; Man: mannose; GalA: galacturonic acid. (0.31 MB PDF) [file pgen.1001388.s005.pdf]

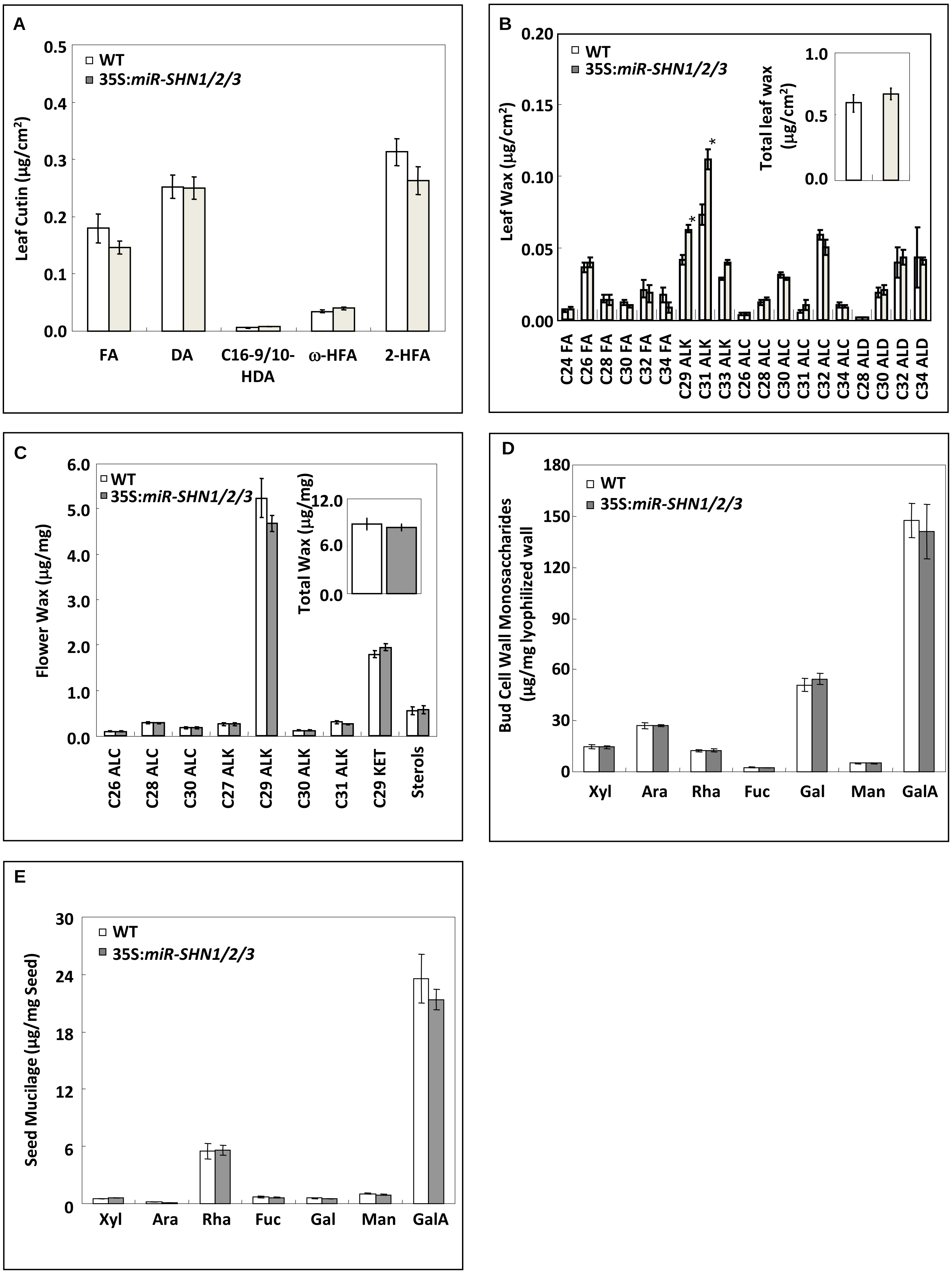

Figure S5. Profiling of leaf cutin, leaf and flower waxes, and bud cell walls and seed mucilage monosaccharides. (A) Cutin profiling of mature rosette leaves. DA, dioic acid monomers; FA, fatty acid; 2-HFA, 2-hydroxy FA; ω-HFA, ω-hydroxy FA; HDA, hydroxy dioic aicds. Values represent means and standard errors (n=3). (B-C) Wax profiling of mature rosette leaves (B) and flowers (C), respectively. Inserted is the total leaf wax. ALC, alcohols; ALD, aldehydes; ALK, alkanes; FA, fatty acid, KET, ketones. Values represent means and standard errors (n=4). *, $p<0.05$. (D-E) Monosaccharide compositions of bud cell walls (D) and seed mucilage (E). Values represent the means and SE (bud: n=5; seed mucilage: n=4). Xyl: xylose; Ara: arabinose; Rha: rhamnose; Fuc: fucose; Gal: galactose; Man: mannose; GalA: galacturonic acid.
