# Supplementary material for: SHINE Transcription Factors Act Redundantly to Pattern the Archetypal Surface of Arabidopsis Flower Organs
Source: PLoS Genet. 2011 May 26;7(5):e1001388. doi: 10.1371/journal.pgen.1001388 (PMC3102738; doi:10.1371/journal.pgen.1001388)
Supplement: Figure S6 — In silico coexpression analysis. (A) Network of SHN1/WIN1 co-expressed genes as revealed by ATTED-II from Tair: http://atted.jp/; Red and green shaded genes represent up-and down-regulated genes in 35S:miR-SHN1/2/3 buds, respectively. (B) Co-correlation scatter plot (2-D Pearson Correlation Coefficients) of some SHN target genes with SHN1 and SHN3, respectively, generated using Arabidopsis Coexpression Data Mining Tools (http://www.arabidopsis.leeds.ac.uk/act). (0.28 MB PDF) [file pgen.1001388.s006.pdf]

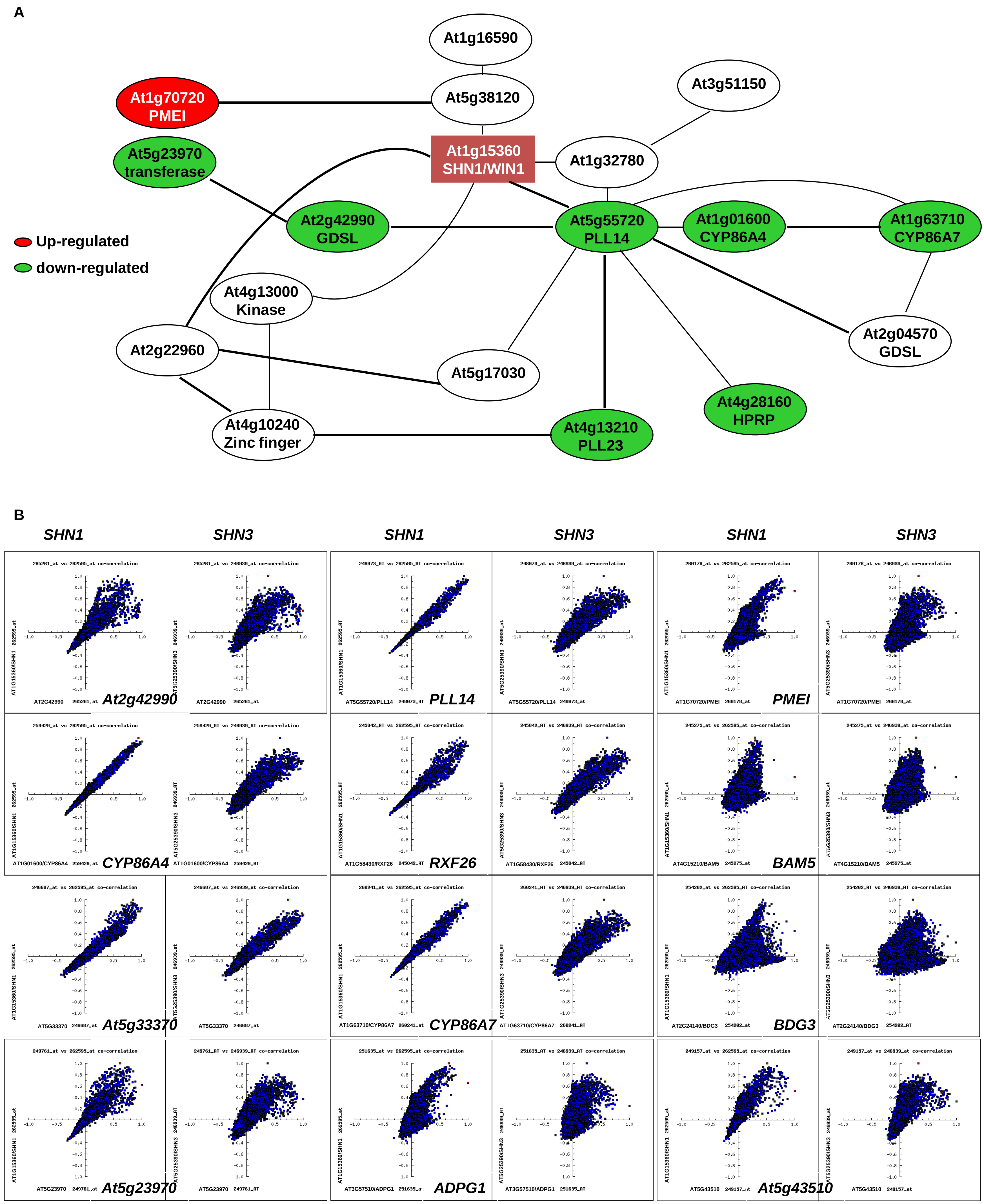


**Figure S6.** ***In silico* coexpression analysis.** (A) Network of *SHN1/WIN1* co-expressed genes as revealed by ATTED-II from Tair (http://atted.jp/). Red and green shaded genes represent up-and down-regulated genes in 35S:*miR-SHN1/2/3* buds, respectively. (B) Co-correlation scatter plot (2-D Pearson Correlation Coefficients) of some *SHN* target genes with both *SHN1* and *SHN3*, respectively, generated using Arabidopsis Coexpression Data Mining Tools (http://www.arabidopsis.leeds.ac.uk/act/).

**Shi et al., Figure S6**
