# Supplementary material for: SHINE Transcription Factors Act Redundantly to Pattern the Archetypal Surface of Arabidopsis Flower Organs
Source: PLoS Genet. 2011 May 26;7(5):e1001388. doi: 10.1371/journal.pgen.1001388 (PMC3102738; doi:10.1371/journal.pgen.1001388)
Supplement: Figure S7 — Genetic modifying putative target genes of SHINE alters petal surface pattering. (A–B) SEM images of the WT petal adaxial and abaxial side, respectively. (C–P) SEM images of petal epidermis (adaxial and abaxial, respectively) derided from knock out plants of At5g23970 (C–D); bdg3 (E–F), At5g03350 (G–H), cyp86a4-1 (I–J), cyp86a4-2 (K–L), at5g33370-1(M–N), at5g33370-2 (O–P). (Q–T) SEM images of petal epidermis (adaxial and abaxial, respectively) derived from artificial microRNA co-silenced CYP86A and GDSL-lipase plants (Q–R, 35S:miR-CYP84A4/A7; S–T, 35S:miR-At5g33370/At3g04290. At3g04290/LTL1 is the closest GDSL lipase to At5g33370 in the same family). (U) Real time RTPCR analysis validation of the downregulation of the expression of both CYP86A4 and CYP86A7 in 35S:miR-CYP86A4/7 plants. (V–W) RT-PCR confirmation of the activation of the microRNA machinery showing the expression of microRNA precursor in various transgenic plant lines. (X) The T-DNA insertion positions of these mutants mentioned above. (0.91 MB PDF) [file pgen.1001388.s007.pdf]

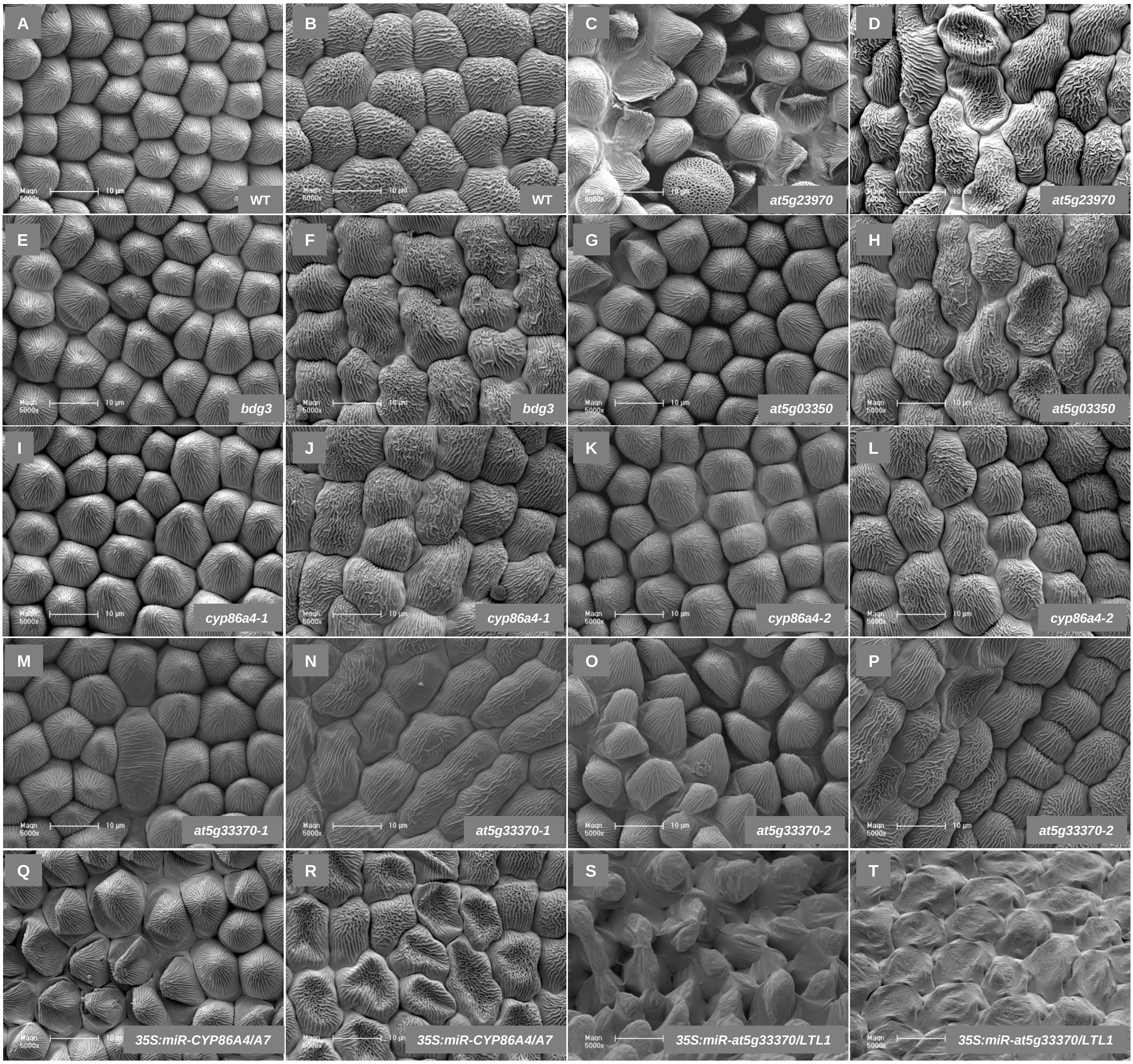

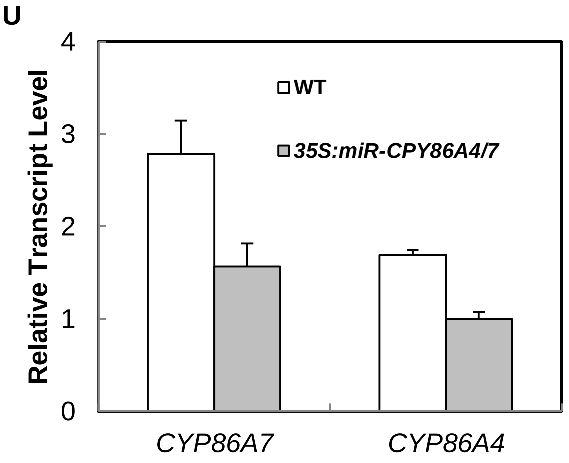

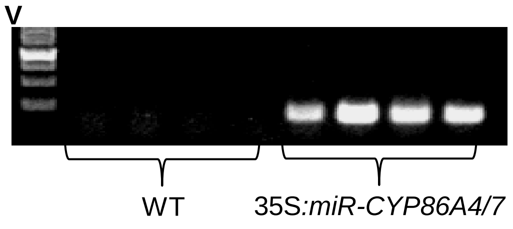

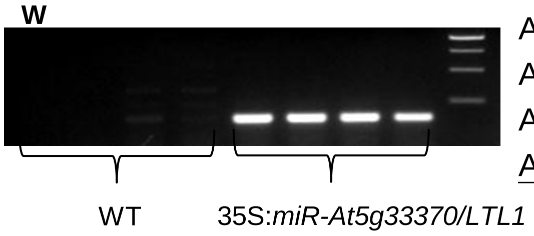

X

Locus	SALK No.	T-DNA Site
AT1G01600	SALK_015303	exon
AT1G01600	SALK_077857C	exon
AT4G24140	SALK_026435C	exon
AT5G23970	SALK_026793C	300-UTR5
AT5G03350	SALK_074760C	300-UTR5
AT5G33370	SALK_032531	promoter
AT5G33370	SALK_070013	300-UTR3

Shi et al., Figure S7

Figure S7. Genetic modifying putative target genes of SHN alters petal surface patterning. (A-B) SEM images of the WT petal adaxial and abaxial side, respectively. (C-P) SEM images of petal epidermis (adaxial and abaxial, respectively) derided from knock out plants of *At5g23970* (C-D); *bdg3* (E-F), *At5g03350* (G-H), *cyp86a4-1* (I-J), *cyp86a4-2* (K-L), *at5g33370-1*(M-N), *at5g33370-2 (*O-P). (Q-T) SEM images of petal epidermis (adaxial and abaxial, respectively) derived from artificial microRNA co-silenced *CYP86A* and *GDSL-lipase* plants (Q-R, 35S*:miR-CYP84A4/A7*; S-T, 35S*:miR-At5g33370/At3g04290*. *At3g04290/LTL1* is the closest GDSL lipase to *At5g33370* in the same family). (U) Real time RT-PCR analysis validation of the downregulation of the expression of both *CYP86A4* and *CYP86A7* in 35S:*miR-CYP86A4/7* plants. (V-W) RT-PCR confirmation of the activation of the microRNA machinery showing the expression of microRNA precursor in various transgenic plant lines. (X) The T-DNA insertion positions of these mutants mentioned above.
