# Supplementary material for: SHINE Transcription Factors Act Redundantly to Pattern the Archetypal Surface of Arabidopsis Flower Organs
Source: PLoS Genet. 2011 May 26;7(5):e1001388. doi: 10.1371/journal.pgen.1001388 (PMC3102738; doi:10.1371/journal.pgen.1001388)
Supplement: Figure S8 — GA regulates the expression of SHN1/WIN1 and several SHINE putative target genes in a DELLA-dependent manner. (A) GA up-regulated SHN1/WIN1 and 13 SHN putative target genes in a DELLA dependent way in the young flower buds. Top panel: GA up-regulated (WT vs. ga1-3); Bottom panel: DELLA up-regulated (penta vs. ga1-3). (B) GA down-regulated 4 SHN putative target genes in a DELLA dependent way in the young flower buds. Top panel: GA down-regulated (WT vs. ga1-3); Bottom panel: DELLA down-regulated (penta vs. ga1-3). All data were adopted from Cao et al., 2006 [5]. Values are means and standard errors (n = 6). ga1-3, loss of function mutant in the GA1 gene which encodes an enzyme involved in GA biosynthesis; penta, GA-deficient quadruple mutant ga1-3 gai-t6 rga-t2 rgl1-1 rgl2-1; In ga1-3, all DELLA proteins are active. (0.18 MB PDF) [file pgen.1001388.s008.pdf]

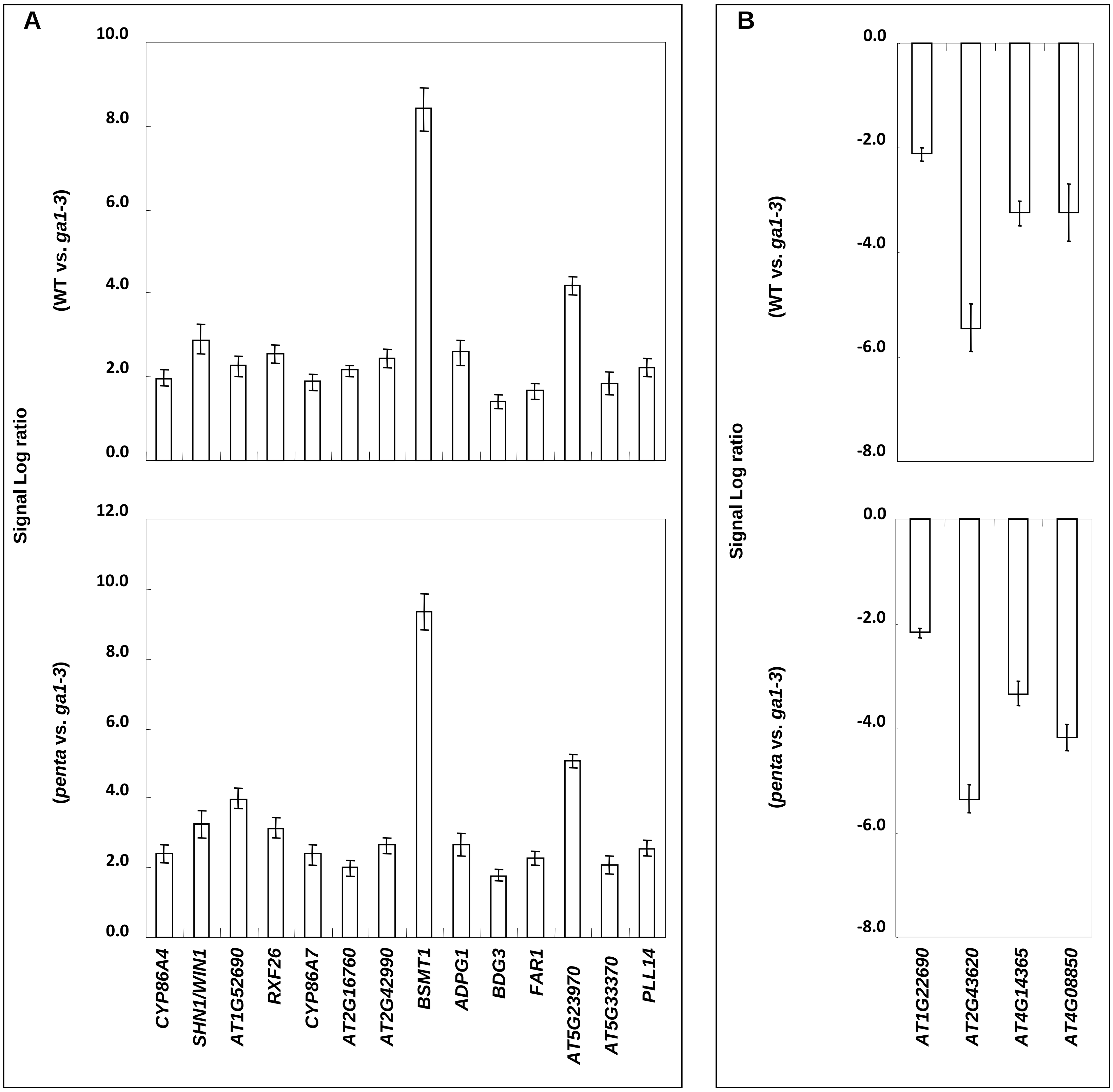

Figure S8. **GA regulates the expression of *SHN1/WIN1* and several SHN putative target genes in a DELLA-dependent manner**. (A) GA up-regulated *SHN1/WIN1* and 13 SHN putative target genes in a DELLA dependent way in the young flower buds. Top panel: GA up-regulated (WT vs. *ga1-3*); Bottom panel: DELLA up-regulated (*penta* vs. *ga1-3*). (B) GA down-regulated 4 SHN putative target genes in a DELLA dependent way in the young flower buds. Top panel: GA down-regulated (WT vs. *ga1-3*); Bottom panel: DELLA down-regulated (*penta* vs. *ga1-3*). All data were adopted from Cao et al., 2006[5]. Values are means and standard errors (n=6). *ga1-3*, loss of function mutant in the GA1 gene which encodes an enzyme involved in GA biosynthesis; *penta*, DELLA quadruple mutant on *ga1-3* background: *ga1-3 gai-t6 rga-t2 rgl1-1 rgl2-1*; In *ga1-3*, all DELLA proteins are active.
