# Supplementary figures and images for: SHINE Transcription Factors Act Redundantly to Pattern the Archetypal Surface of Arabidopsis Flower Organs
Source: PLoS Genet. 2011 May 26;7(5):e1001388. doi: 10.1371/journal.pgen.1001388 (PMC3102738; doi:10.1371/journal.pgen.1001388)

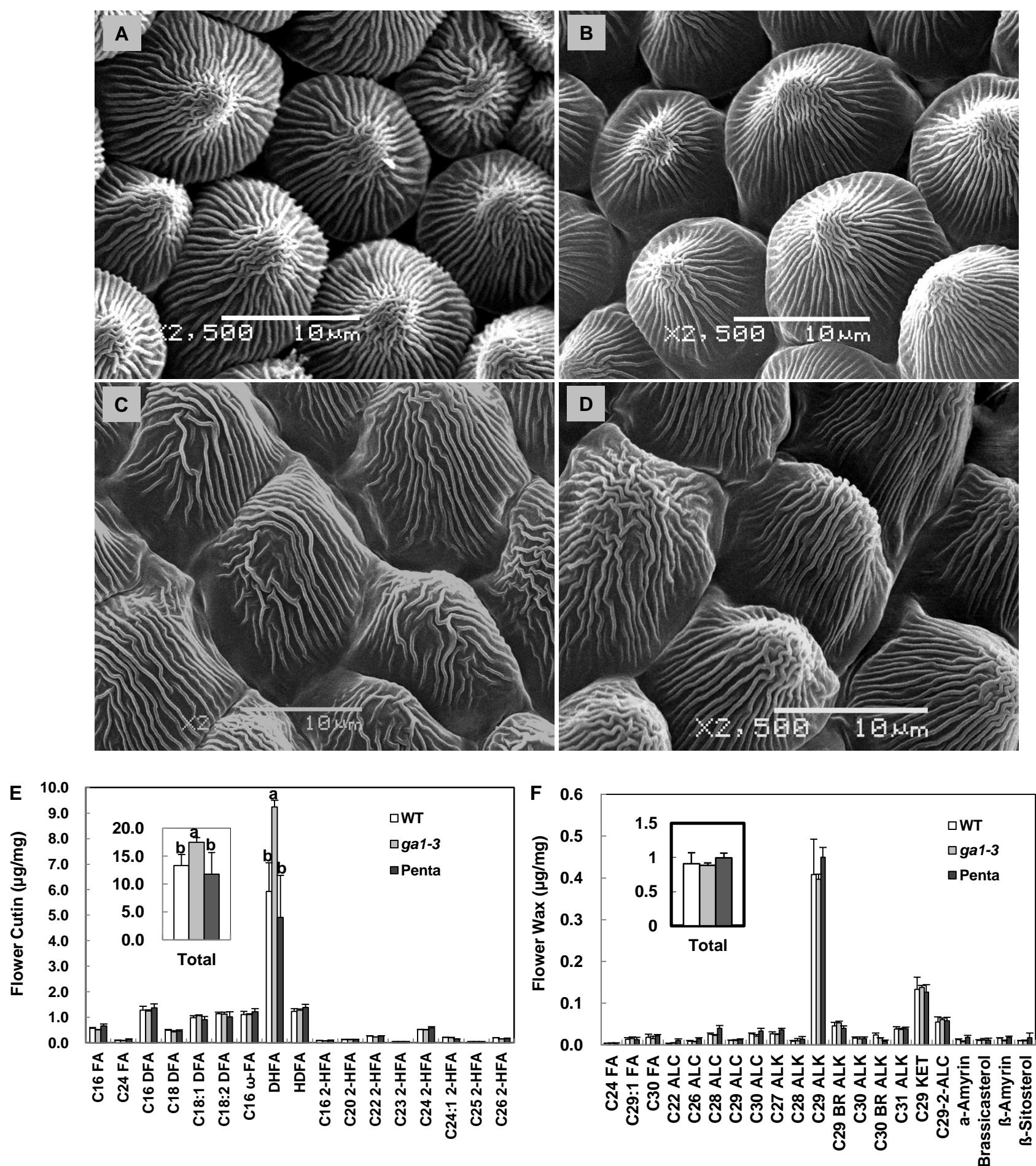

Supplement: Figure S9 — Petal Surface Morphology and Profiling of flower cutin and waxes in GA and or DELLA mutants. (A–D) SEM images of the petal surfaces. A and C, WT adaxial and abaxial petal surface, respectively; B and D, ga1-3 adaxial and abaxial petal surface, respectively. (E) Cutin profiling of open flowers (Inserted is the total cutin). FA, fatty acids; DFA, α,ω-dicarboxylic FA; ω-HFA, ω-hydroxy FA; DHFA, C16/9,10-HFA; HDFA, C16-9/10-hydroxy DFA; 2-HFA, 2-hydroxy FA. Values represent means and standard errors (n = 4). Different letters indicate the significant difference (p<0.05). (F) Wax profiling of open flowers (Inserted is the total wax). FA, fatty acids; ALC, alcohols; ALK, alkanes; BR ALK, branched alkanes; KET, ketones. Values represent means and standard errors (n = 4). (0.66 MB PDF) [file pgen.1001388.s009.pdf]
