# Supplementary material for: SHINE Transcription Factors Act Redundantly to Pattern the Archetypal Surface of Arabidopsis Flower Organs
Source: PLoS Genet. 2011 May 26;7(5):e1001388. doi: 10.1371/journal.pgen.1001388 (PMC3102738; doi:10.1371/journal.pgen.1001388)
Supplement: Figure S10 — Gus expression pattern of SHN3 in the roots and mRNA levels of four SHINE putative target genes in translatomes of different cell populations of Arabidopsis. (A) Gus staining of SHN3 observed in the central cylinder of primary and lateral roots.(B) Cross section through a primary root (maturation zone) showing GUS staining of SHN3 in the parenchymatic cells of the stele. (C–F) Absolute signal values of four putative SHN/WIN target gene transcripts in translatomes isolated from cell populations visualized via the eFP platform (efp.ucr.edu/). (0.25 MB PDF) [file pgen.1001388.s010.pdf]

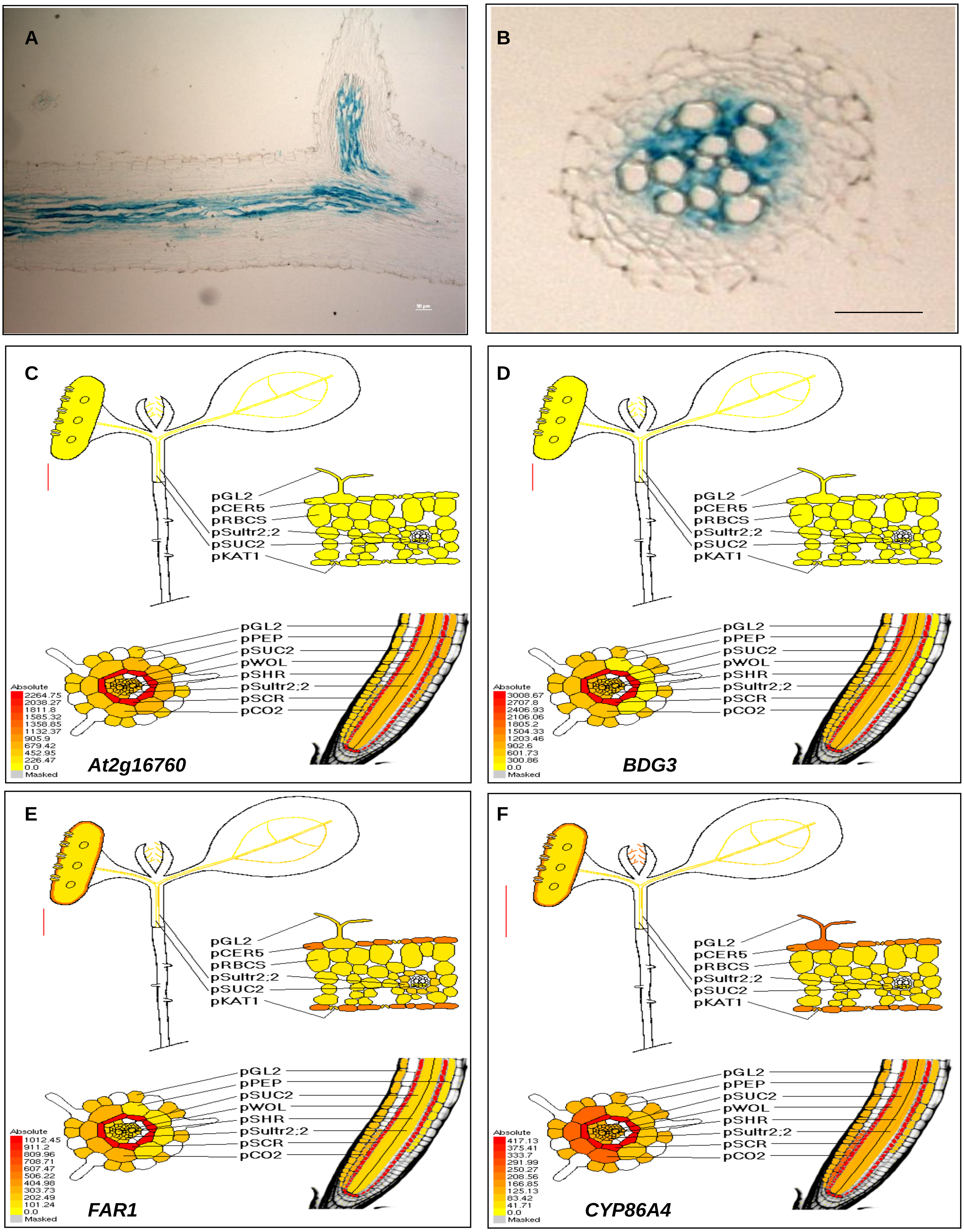

Figure S10. Gus expression pattern of SHN3 in the roots and mRNA levels of four *SHN* putative target genes in translatomes of different cell populations of Arabidopsis. (A) Gus staining of *SHN3* observed in the central cylinder of primary and lateral roots.(B) Cross section through a primary root (maturation zone) showing GUS staining of *SHN3* in the parenchymatic cells of the stele. (C-F) Absolute signal values of four putative *SHN* target gene transcripts in translatomes isolated from cell populations visualized via the eFP platform (efp.ucr.edu/).
