# Supplementary material for: SHINE Transcription Factors Act Redundantly to Pattern the Archetypal Surface of Arabidopsis Flower Organs
Source: PLoS Genet. 2011 May 26;7(5):e1001388. doi: 10.1371/journal.pgen.1001388 (PMC3102738; doi:10.1371/journal.pgen.1001388)
Supplement: Text S1 — Supporting Materials and Methods and References. (0.04 MB DOC) [file pgen.1001388.s013.doc]

**Supporting M&M and References**

**Material and Methods**

**RACE analysis of cleaved miRNA target genes**

Cleavage sites in the miRNA target genes were mapped using RLM-RACE, a modified 5' RACE procedure as described by [1] using the GeneRacer (Invitrogen) protocol coupled with nested gene-specific primers
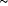
200 to 400 nucleotides downstream of the predicted miRNA target site. The PCR products were purified and directly sequenced or cloned into pGEMT Easy and sequenced.

**Seed mucilage extraction, bud cell wall isolation, and TMS GC-MS analysis**

Seed mucilage and bud cell wall isolation were performed according to [2] and [3], respectively, and wall materials were hydrolyzed for 2 h at 120^o^C in 2 M trifluoroacetic acid [4]. Hydrolysates were lyophilized, derivatized with methoxyamine hydrochloride (20 g/ml in pyridine) for 1.5 h at 37^o^C, and trimethylsilated with N-Methyl-N-trifluoroacetamide for 30 min at 37^o^C. One μl of the resulting trimethylsilates were injected onto TRACE GC ULTRA (Thermo Electron Corporation). Ribose (used as internal standard), neutral sugar standards (fucose, arabinose, rhamnose, galactose, mannose, xylose), and acid sugar (galacturonic acid) were also processed in this way. Each monosaccharide was quantified using its standard curve established with monosaccharide standards of different concentrations in relation to the same concentration of ribose.

**Quantitative real time RT-PCR (qRT-PCR) analysis**

A certain amount of DNAse-treated total RNA was reverse transcribed using the AMV Reverse Transcriptase (EurX Ltd., Poland). qRT-PCR gene expression analysis was performed in three biological replicates using gene-specific qRT-PCR oligonucleotides (Table S2) designed with the Primer Express software (Applied Biosystems). RT-PCR reactions were tracked on an ABI 7300 instrument (Applied Biosystems) using the PlatinumR SYBR SuperMix (Invitrogen). Each sample was PCR-amplified from the same amount of cDNA template in triplicate reactions. Following an initial step in the thermal cycler for 15 min. at 95^o^C, PCR amplification proceeded for 40 cycles of 15 s. at 95^o^C and 30 s. at 60^o^C, and completed by melting curve analysis to confirm specificity of the PCR products. The baseline and threshold values were adjusted according to the manufacturer’s instructions.

**References**

1. Alvarez JP, Pekker I, Goldshmidt A, Blum E, Amsellem Z, et al. (2006). Endogenous and synthetic microRNAs stimulate simultaneous, efficient, and localized regulation of multiple targets in diverse species. Plant Cell 18: 1134-1151.

2. Western TL, Skinner DJ, Haughn GW (2000) Differentiation of mucilage secretory cells of the Arabidopsis seed coat. Plant Physiol 122: 345-355.

3. Caffall KH, Pattathil S, Phillips SE, Hahn MG, Mohnen D (2009) Arabidopsis thaliana T-DNA mutants implicate *GAUT* genes in the biosynthesis of pectin and xylan in cell walls and seed testa. Mol Plant 2: 1000-1014.

4. Arnous A, Meyer AS (2009) Quantitative prediction of cell wall polysaccharide composition in grape (*Vitis vinifera* L.) and apple (*Malus domestica*) skins from acid hydrolysis monosaccharide profiles. J Agri Food Chem 57: 3611-3619.

5. Cao DN, Cheng H, Wu W, Soo HM, Peng JR (2006) Gibberellin mobilizes distinct DELLA-dependent transcriptomes to regulate seed germination and floral development in Arabidopsis. Plant Physiol 142: 509-525.

**Supplemental Table Titles**

**Table1 S1.** Lists of primers used in this study**.**

**Table1 S2.** List of genes co-expressed with *SHN1/WIN1* or *SHN3* analyzed with GeneCat (http://genecat.mpg.de/)
